# Hematological and renal toxicity in mice after three cycles of high activity [^177^Lu]Lu-PSMA-617 with or without human α_1_-microglobulin

**DOI:** 10.1038/s41598-024-61370-2

**Published:** 2024-05-11

**Authors:** Amanda Kristiansson, Oskar Vilhelmsson Timmermand, Mohamed Altai, Sven-Erik Strand, Bo Åkerström, Anders Örbom

**Affiliations:** 1 Department of Clinical Sciences Lund, Section for Oncology, Lund University, Barngatan 4, 222 42 Lund, Sweden; 2 Department of Clinical Sciences Lund, Section for Pediatrics, Lund University, Lund, Sweden; 3 Department of Neonatology, Skåne University Hospital, Lund, Sweden; 4 Department of Imaging Chemistry and Biology, School of Biomedical Engineering and Imaging Sciences, King’s College London, London, UK; 5 Department of Clinical Sciences Lund, Section for Medical Radiation Physics, Lund University, Lund, Sweden; 6 Department of Clinical Sciences Lund, Section for Infection Medicine, Lund University, Lund, Sweden

**Keywords:** Targeted therapies, Cancer models, Prostate cancer, Kidney

## Abstract

Radioligand therapy with [^177^Lu]Lu-PSMA-617 can be used to prolong life and reduce tumor burden in terminally ill castration resistant prostate cancer patients. Still, accumulation in healthy tissue limits the activity that can be administered. Therefore, fractionated therapy is used to lower toxicity. However, there might be a need to reduce toxicity even further with e.g. radioprotectors. The aim of this study was to (i). establish a preclinical mouse model with fractionated high activity therapy of three consecutive doses of 200 MBq [^177^Lu]Lu-PSMA-617 in which we aimed to (ii). achieve measurable hematotoxicity and nephrotoxicity and to (iii). analyze the potential protective effect of co-injecting recombinant α_1_-microglobulin (rA1M), a human antioxidant previously shown to have radioprotective effects. In both groups, three cycles resulted in increased albuminuria for each cycle, with large individual variation. Another marker of kidney injury, serum blood urea nitrogen (BUN), was only significantly increased compared to control animals after the third cycle. The number of white and red blood cells decreased significantly and did not reach the levels of control animals during the experiment. rA1M did reduce absorbed dose to kidney but did not show significant protection here, but future studies are warranted due to the recent clinical studies showing a significant renoprotective effect in patients.

## Introduction

Metastatic castration resistant prostate cancer (CRPC) is one of the leading cancer-related causes of mortality in men^1^. Androgen ablation is the first-line choice of treatment in metastatic prostate cancer as prostatic tissue is dependent on androgens for growth. This therapy is usually effective short term, but most patients undergoing androgen deprivation therapy progress into CRPC and relapse.

Radioligand therapy (RLT) has become a powerful cancer therapy treatment alternative^2^. In prostate cancer, RLT and radioligand imaging has been developed towards prostate-specific membrane antigen (PSMA), a transmembrane protein expressed on most prostate cancer cells^3^. PSMA targeted imaging correlates with blood prostate specific antigen levels and Gleason score^4,5^. PSMA expression has also been shown to be higher in aggressive prostate cancer^6^. Further, PSMA RLT, using Lutetium-177 labeled PSMA-617 ([^177^Lu]Lu-PSMA-617) prolong life and reduce tumor burden in advanced stage CRPC patients^7–9^. The phase 3 VISON trial showed that [^177^Lu]Lu-PSMA-617 together with standard-of-care prolonged the median radiological progression free survival with 5.3 months and the overall survival with 4 months compared to standard-of-care only^10^. Following this outcome, [^177^Lu]Lu-PSMA-617 was approved in 2022 by the FDA and EMA as treatment in PSMA-positive metastatic CRPC.

PSMA is also expressed in non-prostate tissue, e.g. the proximal tubule of the kidney, the small intestines, and the salivary glands^11,12^. Accumulation of [^177^Lu]Lu-PSMA-617 in healthy tissue limits the activity that can be administered to patients. Most of the injected activity is found in renal tissue within 10–30 min after injection^3^. In patients treated with [^177^Lu]Lu-PSMA-617, kidneys have received an absorbed dose up to 30 Gy with only mild nephrotoxicity. Nephrotoxicity, however, has been seen after extensive, absorbed dose of 40–50 Gy to the kidneys, PSMA RLT^13^. The activity injected during RLT is often limited by acute hematotoxicity, so to increase the total absorbed dose to the tumor, the therapy is delivered in cycles^14^. For the VISION trial, the cycles were administered 6 weeks apart^10^. Hematological adverse events have been reported, e.g., two studies reported grade 3–4 hematotoxicity in 12–13% of the patients^15,16^. Increasing tumor absorbed dose by increasing administered activity might mean exceeding the recommended kidney absorbed dose limit and lead to potentially permanent renal damage as well as a higher frequency of hematotoxicity, which can present a challenge if RLT is proposed for earlier stage disease with longer projected survival^17^.

Human α_1_-microglobulin (A1M) is a naturally occurring radical scavenger, heme binder, reductase and antioxidant^18^. Studies show that recombinant human A1M (rA1M) has renoprotective effects in peptide receptor radionuclide therapy (PRRT) mouse models with [^177^Lu]Lu-DOTATATE^19^, without affecting the tumor treatment efficacy^20^. Furthermore, A1M binds to peripheral white blood cells via specific receptors^21^, protects against hemolysis^22^ and was shown to have hematoprotective effects in mice injected with [^177^Lu]Lu-DOTATATE^23^. For [^177^Lu]Lu-PSMA-617 rA1M has been shown not affect the tumor treatment, and there are indications that it may preserve kidney function^24^. A1M may therefore be a potential renoprotective and hematoprotective agent during [^177^Lu]Lu-PSMA-617 RLT prostate cancer treatment. It is important to note that the proposed radioprotective mechanism of rA1M is different to that of co-administration of an amino acid solution which is done routinely during PRRT^25^. Co-infused amino acids blocks kidney uptake of the radioligand, lowering kidney absorbed dose, while radical scavengers like rA1M is suggested to prevent radical and oxidative damage to e.g. DNA^26^.

In order to reach absorbed doses to the kidney that causes measurable damage, we have previously established that mice can recover from hematotoxicity caused by one injection of up to 200 MBq of [^177^Lu]Lu-PSMA-617^27^. The aim of this study was to employ a high activity therapy delivered in three cycles of [^177^Lu]Lu-PSMA-617 in a preclinical mouse model to achieve nephrotoxicity and analyze the effect of co-injecting rA1M on kidney function and blood cell counts.

## Results

### Labeling of PSMA-617 with ^177^Lu and ^111^In

Labeling of PSMA-617 resulted in 99 ± 1% and 98 ± 0.5% radiochemical yield for ^177^Lu and ^111^In, respectively. [^177^Lu]Lu-PSMA-617 specific activity was 66.6 MBq/nmol molecule and [^111^In]In-PSMA-617 was 3.2 MBq/nmol molecule. Both conjugates, [^177^Lu]Lu-PSMA-617 and [^111^In]In-PSMA-617 required no further purification before administration into animals due to the high achieved radiochemical yield.

### Animals co-injected with rA1M receive lower absorbed dose to kidney

**Figure 1:**
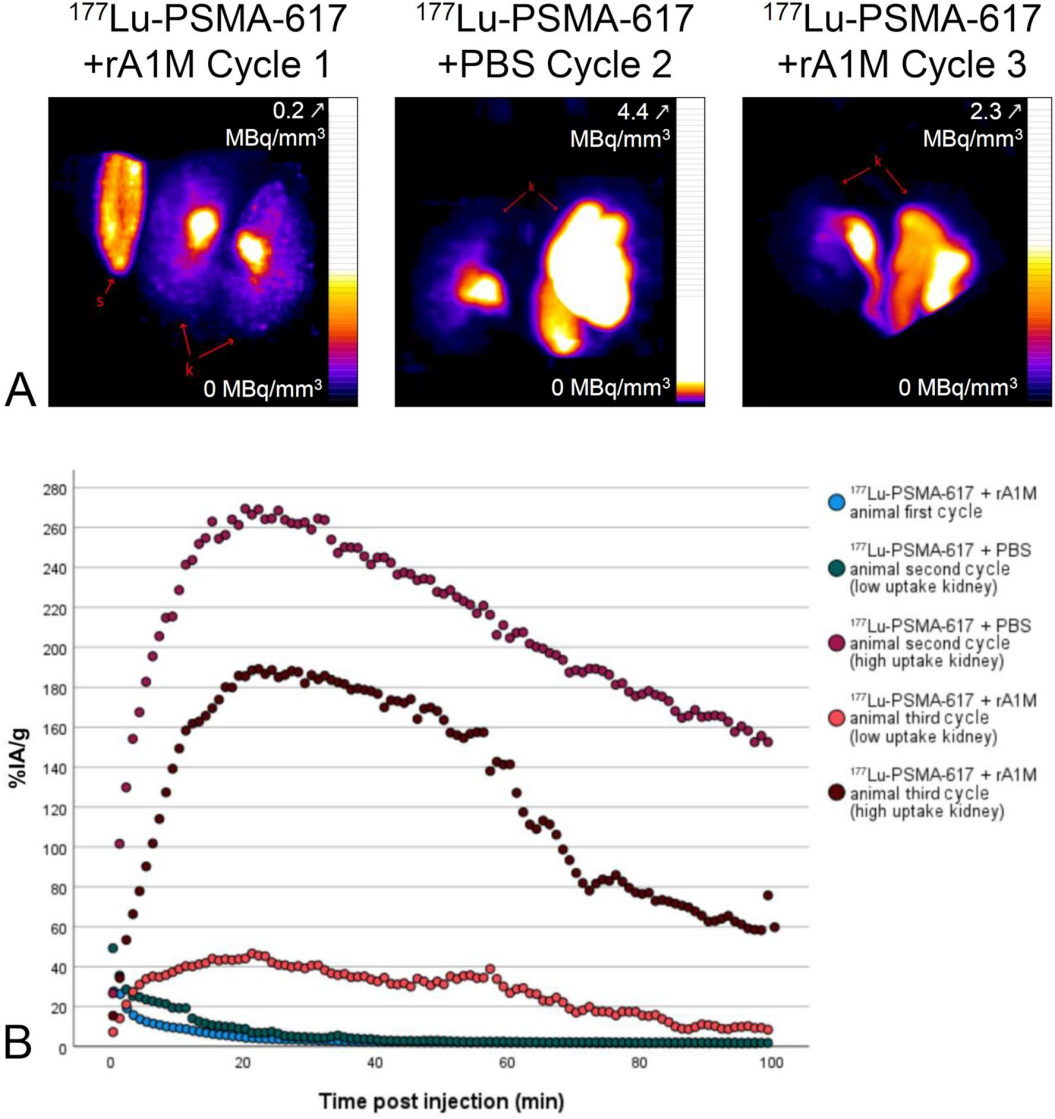
Uptake characteristics of [^177^Lu]Lu-PSMA-617 changes after each cycle of treatment*.* Representative SPECT images and graphs of [^177^Lu]Lu-PSMA-617 activity in male BALB/cAnNRj nude mice. Maximum intensity projections using the NIH color scale windowed to best display kidneys with the top activity noted. Note that the Cycle 2 animal is the one with an outlier kidney. Annotations: k = kidney, and s = standard. (**A**) Sum of all frames of the dynamic scans immediately p.i. with 90–100 frames of 1 min. (**B**) Examples of [^177^Lu]Lu-PSMA-617 kinetics in kidneys as measured using dynamic SPECT imaging directly after injection. Percent of injected activity per gram of kidney (%IA/g) plotted. Chosen animals have typical distribution for their cycles except for the second cycle animal where the high uptake kidney is an outlier.

For cycle 1, only one kidney was quantified per animal, for cycle 2 one [^177^Lu]Lu-PSMA-617 + PBS animal had both quantified, and for cycle 3, both were quantified for two [^177^Lu]Lu-PSMA-617 + rA1M animals and two [^177^Lu]Lu-PSMA-617 + PBS animals. Representative SPECT images can be seen in Fig. 1A and supplementary Figure S1. In the initial kidney accumulation, all animals exhibited a very quick accumulation and excretion when imaged during cycle 1, while some exhibited higher and slower accumulation during the second cycle imaging, and many more did so during the third cycle (exemplified in Fig. 1B). Supplementary Figure S2 shows the amount of decays calculated in measured kidneys for the first 48 h, note the outlier [^177^Lu]Lu-PSMA-617 + PBS kidney in cycle 2. No statistically significant difference between groups was found for the accumulated number of decays using the Mann–Whitney U test (*p*-values > 0.485). The absorbed dose per injected activity value calculated for each group was applied to the injected activities and the resulting absorbed doses for each cycle can be seen in Table 1.

**Table 1:** Animals co-injected with rA1M receive lower absorbed dose to kidney. Mean injected activity, excluding any activity extravasated in the tail at time of imaging.

	Cycle 1	Cycle 2	Cycle 3	Total
Mean injected activity (MBq)				
[^177^Lu]Lu-PSMA-617 + rA1M	183.6	191.5	183.3	558.4
[^177^Lu]Lu-PSMA-617 + PBS	182.9	185.7	194.4	563
Kidney absorbed dose per injected activity (Gy/MBq)				
[^177^Lu]Lu-PSMA-617 + rA1M	0.052	0.053	0.152	
[^177^Lu]Lu-PSMA-617 + PBS	0.045	0.037 (0.161*)	0.209	
Mean kidney absorbed dose (Gy)				
[^177^Lu]Lu-PSMA-617 + rA1M	9.60	10.29	27.78	45.35
[^177^Lu]Lu-PSMA-617 + PBS	8.247	6.78 (29.80*)	40.60	55.63 (78.70*)

*Including outlier kidney in cycle 2.

Absorbed dose per injected activity and resulting absorbed dose are each calculated 5 weeks p.i. Note that the values differ substantially whether an outlier kidney in cycle 2 is included or not, thus both results are included.

**Figure 2:**
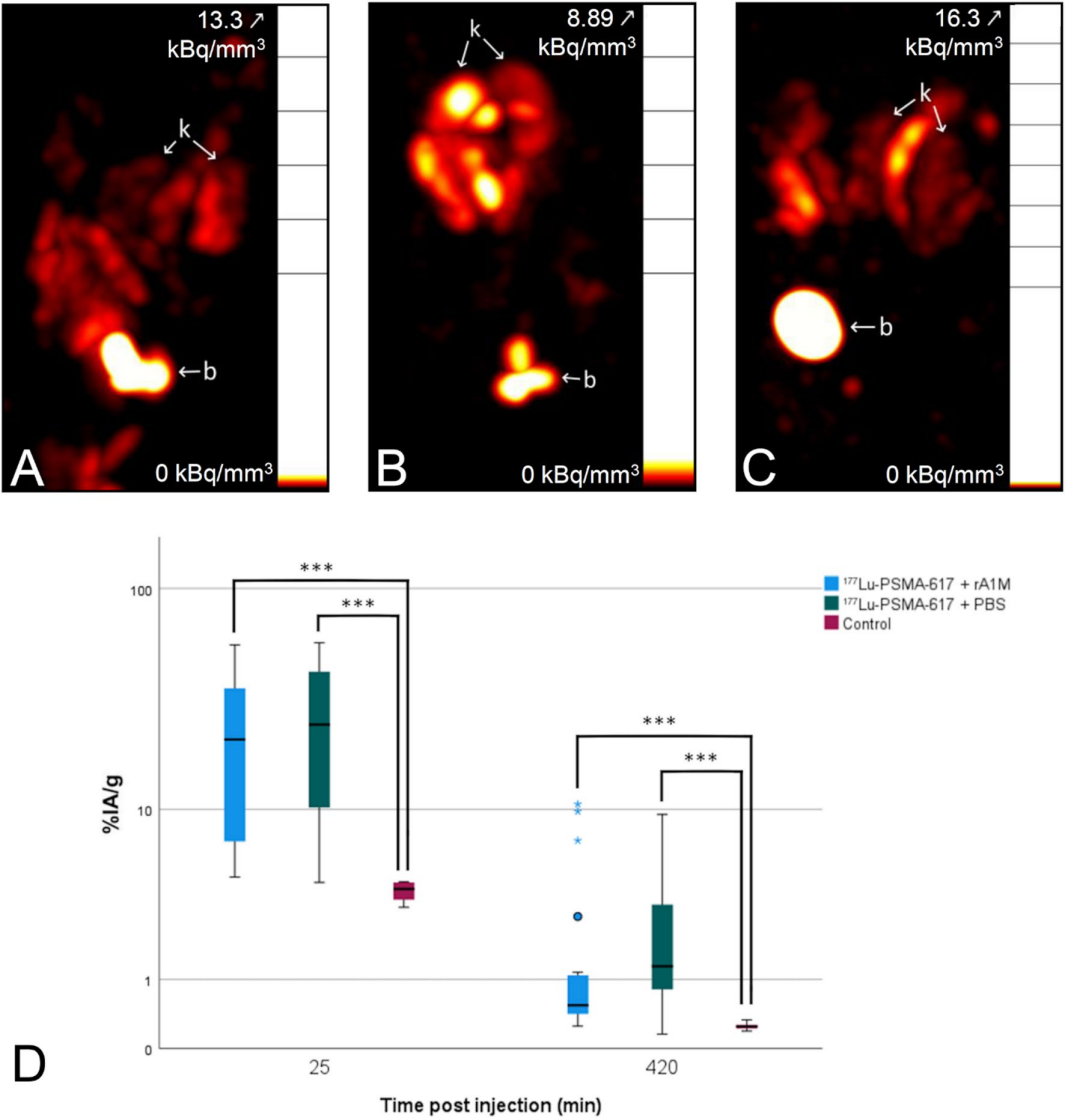
Different uptake of [^111^In]In-PSMA-617 between treated and untreated animals but not between animals co-injected with rA1M or not. SPECT images of the ^111^In activity in animals convolved by a 2 mm FWHM Gauss filter, taken 7 h p.i. [^111^In]In-PSMA-617 at 135 days post first ^177^Lu injection. Maximum intensity projections using the “Hot” color scale windowed to best display kidneys with the top activity noted. Annotations: b = bladder and k = kidney. (**A**) Animal from the [^177^Lu}Lu-PSMA-617 + rA1M group. (**B**) Animal from the [^177^Lu}Lu-PSMA-617 + PBS group. Outlier in cycle 2. (**C**) Animal from the untreated control group. (**D**) Kidney accumulation (%IA/g) of [^111^In]In-PSMA-617 at end of study 135 days post first injection. The [^177^Lu]Lu-PSMA-617 + PBS group had higher mean and median accumulation than [^177^Lu]Lu-PSMA-617 + rA1M, at both time points, the difference, however, was not statistically significant. Both PSMA groups had significantly higher accumulation than the untreated group using the Kruskal–Wallis test and the Bonferroni correction for multiple tests. ★ indicates values labelled as major outliers by the statistical analysis software SPSS. ***p < 0.001.

The post-cycle 3 imaging session at 135 days after the first injection revealed that the [^177^Lu]Lu-PSMA-617 + PBS group had higher mean and median accumulation than [^177^Lu]Lu-PSMA-617 + rA1M, see Fig. 2D, at both time points. This indicates a slight protection effect from rA1M, but the difference was not statistically significant (*p*-values > 0.099). Both PSMA groups had significantly higher accumulation than the untreated group (*p*-values < 0.001) using the Kruskal–Wallis test and the Bonferroni correction for multiple tests. Representative SPECT images can be seen in Fig. 2A–C.

### Non-transient hematotoxicity in all [^177^Lu]Lu-PSMA-617 treated animals

**Figure 3:**
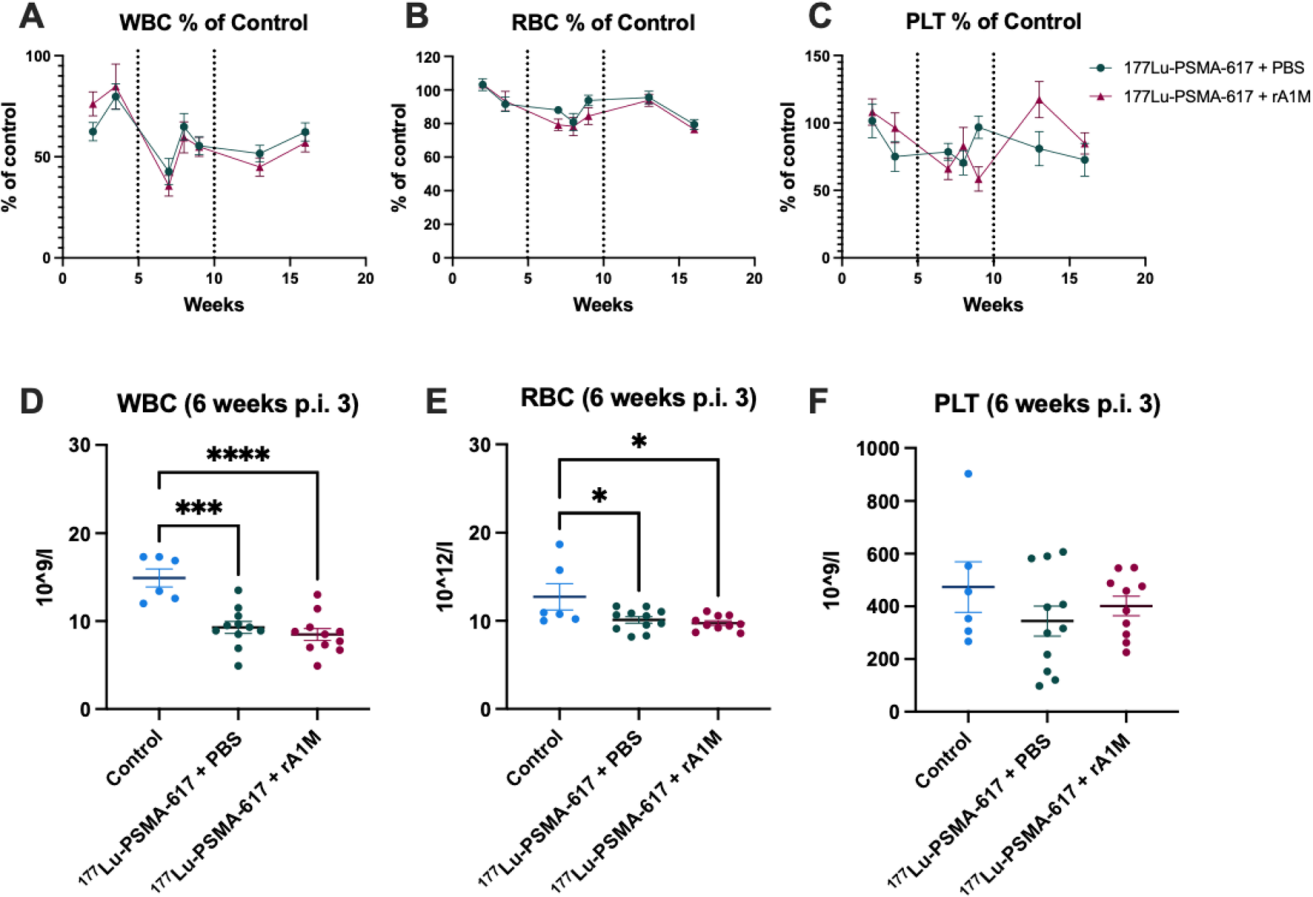
Animals treated with [^177^Lu]Lu-PSMA-617 had a decrease in blood cell counts. Blood cell counts during the course of the experiment. Percent of WBC (**A**), RBC (**B**) and PLT (**C**) compared to control animals. Number of WBC (**D**), RBC (**E**), and PLT (**F**) 6 weeks after last cycle. Dotted line represents when new cycle where administered. Data are presented as line charts (**A**–**C**) or scatter plots with mean (± SEM) (**D**–**F**). Statistical Statistical comparison between groups was made with one-way ANOVA with a Tukey's multiple comparisons post hoc test (**D**–**F**). Only significant differences are presented in the figure. *p < 0.05, *** p < 0.001, **** p < 0.0001.

The white blood cell counts (WBC) showed a significant drop 2 weeks after the first cycle in the [^177^Lu]Lu-PSMA-617 + PBS group that recovered after 3.5 weeks (Fig. 3A, supplementary S3–S4). The drop after the second cycle was significant in both groups compared to control mice. The WBC values did not reach the control levels again, neither after the second cycle nor after the third cycle (Fig. 3A, D). The RBC did not decrease significantly in the [^177^Lu]Lu-PSMA-617 + PBS or [^177^Lu]Lu-PSMA-617 + rA1M group compared to control group at either of the timepoints post-injection one (Fig. 3B, supplementary S3–S4). However, the [^177^Lu]Lu-PSMA-617 + rA1M group decreased significantly compared to control mice after the second cycle but recovered 4 weeks after (Supplementary Figure S5–S7). The third cycle resulted in a significantly lower levels of RBC after six weeks (Fig. 3B, D) in both groups receiving [^177^Lu]Lu-PSMA-617. The platelet values were more stable throughout the experiments for all three cycles, the only significantly lower levels were detected 4 weeks after second cycle (Fig. 3C, F, supplementary S7). All blood cell counts can be found in the supplementary materials (Supplementary Figure S3–S9).

### Animals treated with [^177^Lu]Lu-PSMA-617 had elevated albumin in urine, serum BUN and lower weight gain

**Figure 4:**
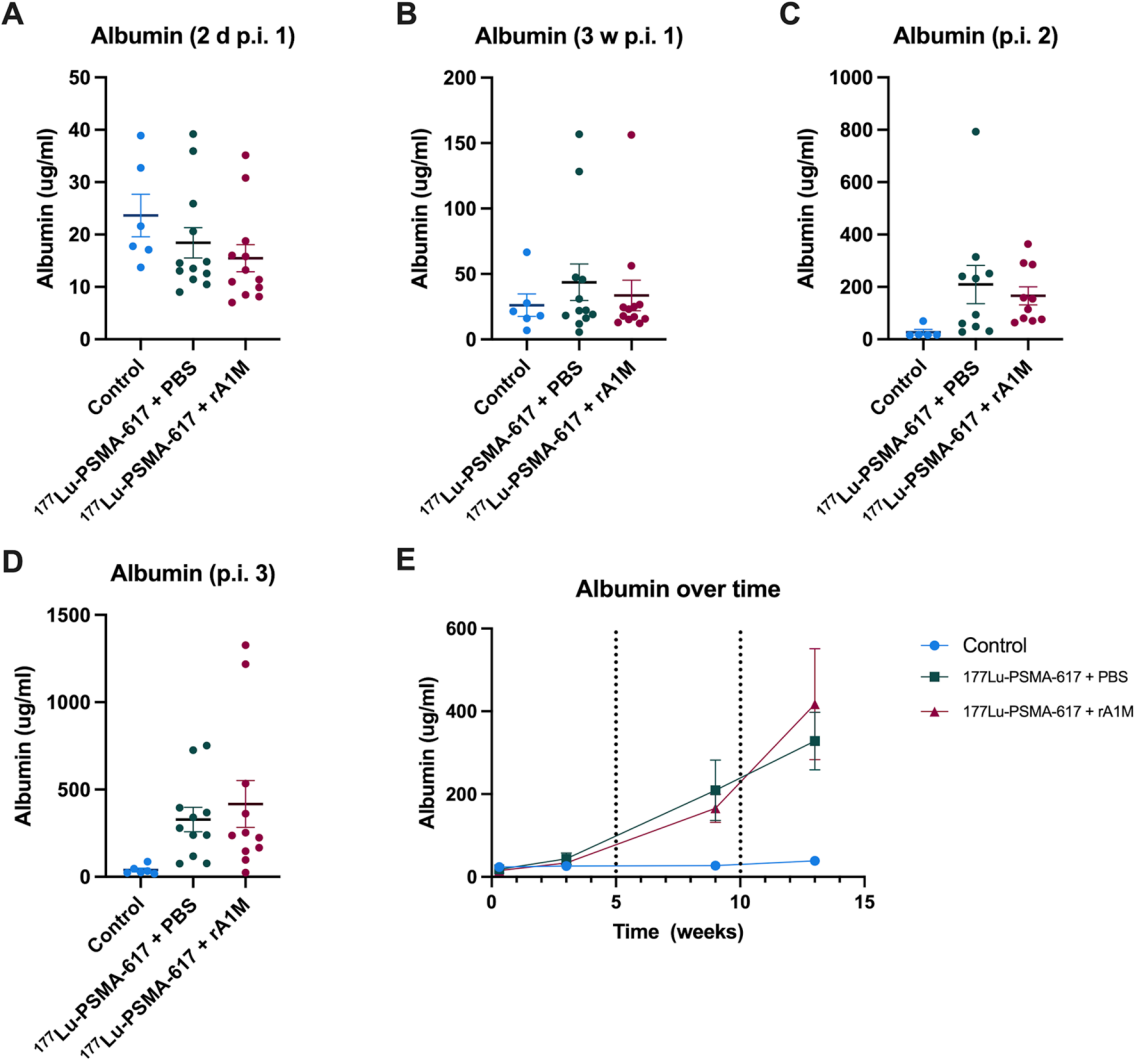
Animals treated with [^177^Lu]Lu-PSMA-617 had elevated albumin in urine. Albumin levels in urine 2 days after first cycle (**A**), three weeks after first cycle (**B**). after second cycle (**C**), after third cycle (**D**) and over time (**E**). Data are presented as scatter plots with mean (± SEM). Statistical Statistical comparison between groups was made with one-way ANOVA with a Tukey's multiple comparisons post hoc test.

**Figure 5:**
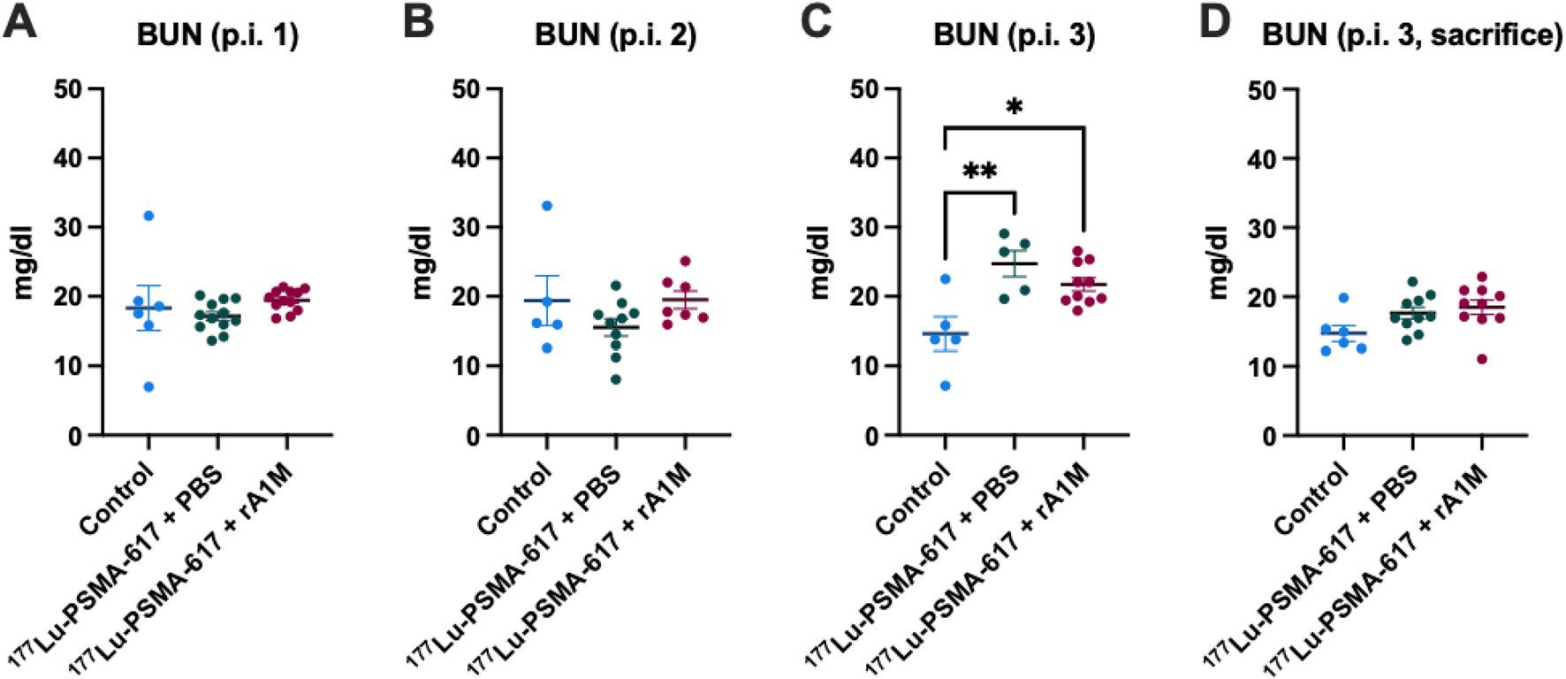
Animals treated with [^177^Lu]Lu-PSMA-617 had elevated serum BUN levels after three cycles. BUN serum levels after first cycle (**A**), after second cycle (**B**). after third cycle (**C**), after third cycle before sacrifice (**D**). Data are presented as scatter plots with mean (± SEM). Statistical comparison between groups was made with one-way ANOVA with a Tukey's multiple comparisons post hoc test. Only significant differences are presented in the figure. *p < 0.05, **p < 0.01.

**Figure 6:**
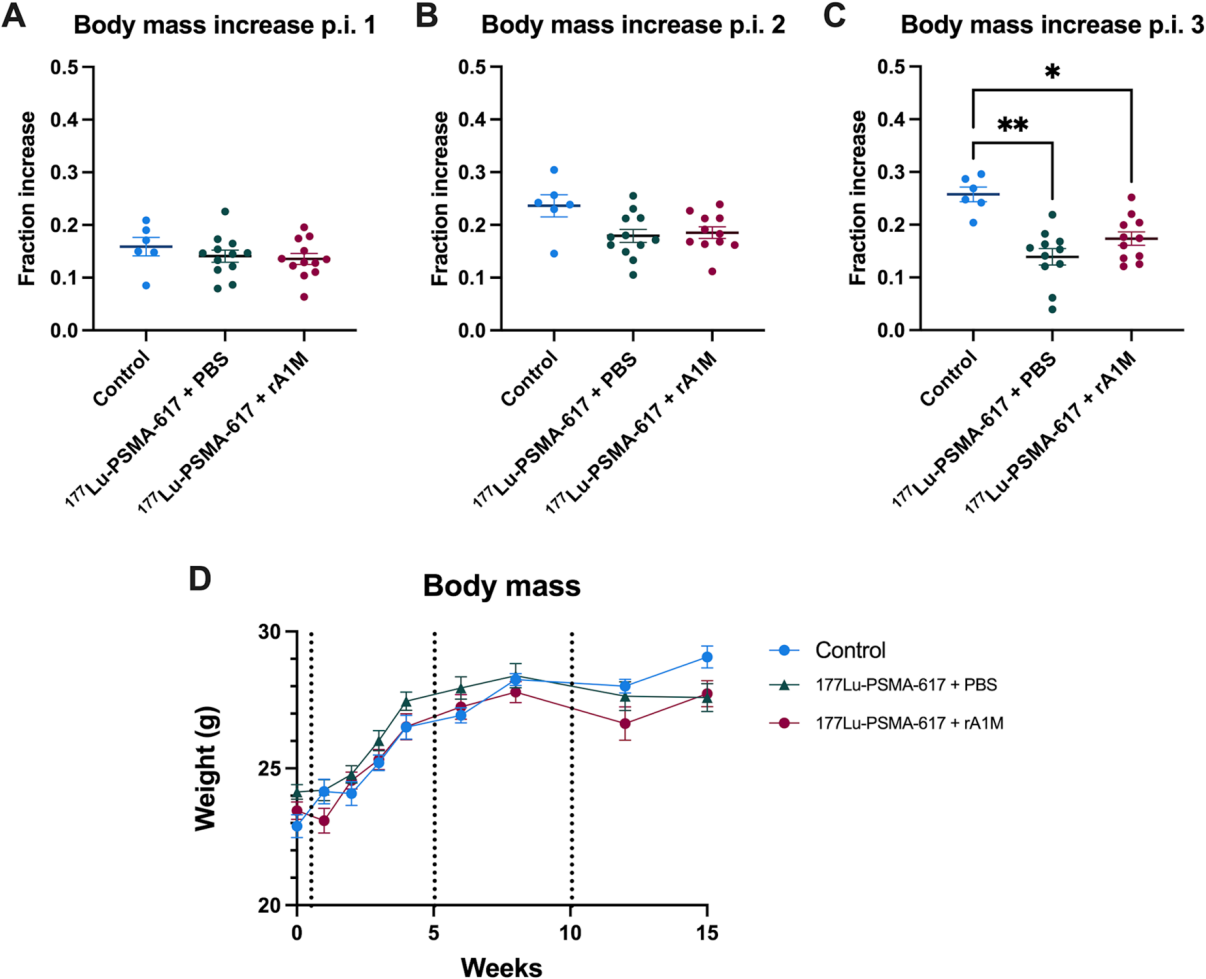
Animals treated with [^177^Lu]Lu-PSMA-617 had lower weight gain. Changes in body mass (calculated as fraction body mass increase; body mass increase/baseline body mass) after 1 (**A**), 2 (**B**) and 3 cycles (**C**). Body mass presented as group mean (± SEM) during the experiment (**D**), the dotted lines represent each cycle of [^177^Lu]Lu-PSMA-617 injected. Data are presented as scatter plots with mean (± SEM). Statistical comparison between groups (**A**–**C**) was made with Kruskal–Wallis test (Dunn’s multiple comparison test). Only significant differences are presented in the figure. *p < 0.05, ** *p* < 0.01.

To monitor the effect of each cycle on renal function, urine albumin was measured after each cycle (Fig. 4). Although no significant differences were seen between either control, [^177^Lu]Lu-PSMA-617 + PBS and [^177^Lu]Lu-PSMA-617 + rA1M groups, there is a clear increase of animals with albumin leakage for each cycle. The levels, however, varies greatly in both groups exposed to [^177^Lu]Lu-PSMA-617. To account for differences in urine dilution, creatinine was measured for available samples, resulting in a significant increase of albumin/creatinine ratio in the [^177^Lu]Lu-PSMA-617 + PBS group compared to control group (Supplementary Figure S10). Moreover, serum BUN was measured after each cycle and before sacrifice (Fig. 5). Levels of BUN was only significantly increased after the third cycle, compared to control animals. However, no difference was detected between [^177^Lu]Lu-PSMA-617 + PBS and [^177^Lu]Lu-PSMA-617 + rA1M groups. To further assess the kidneys, histological examination by counting viable glomeruli was performed in five random animals from each group. However, there were no differences in the number of glomeruli between the groups (Supplementary Figure S11). The weight of the mice was observed during the experiment. After the first two cycles, a similar increase in body mass was seen in all three groups. However, at the time for sacrifice the two groups that received [^177^Lu]Lu-PSMA-617 had a significantly lower increase in body mass (calculated as fraction gain from starting weight, Fig. 6).

## Discussion

Three cycles of high activity injections gave non-transient effects of radiotoxicity to the animals, measurable through increased albuminuria, a clear body weight deficit and lower numbers of circulating WBC and RBC. A significant protective effect of co-injecting rA1M could not be shown in our experimental setup, although some radioprotective effect was indicated by the lower absorbed dose. However, this model with multiple cycles of [^177^Lu]Lu-PSMA-617 could be further used to study the resulting toxicity and strategies to diminish side effects with different interventions. We chose nude mice as our animal model, even though xenograft tumors were not implanted, mainly so our findings would be relevant for future studies that include tumors. It should be noted that nude Balb/c mice differ hematologically from their wild type counterparts, e.g. in lower WBC values^28^. At these high injected activities, the amount of injected ligand is also high and might not be comparable to studies using smaller injected amounts. Similar studies that used lower injected activities of [^177^Lu]Lu-PSMA-617 did not find any significant difference in hematological measurements between treated animals and untreated controls, Tschan et al. at 30 MBq^29^, or between co-injection of rA1M or PBS, our previous study at 100 MBq^24^.

The absorbed doses to the kidneys calculated during this study are by necessity an approximation. The dynamic imaging immediately post injection, necessary due to variation in pharmacokinetics over time in the study, limited the number of animals per cycle, and thereby also the statistical accuracy. SPECT quantification was done by hand by a single operator, but there may still be variation between animals. Quantified accumulation per gram was used with a simulated kidney of a specific size for dosimetry since S-values are organ size-specific^30^. Both full kidneys could not always be in the field of view due to the difficulty of locating them on a low-dose CT, which could lead to over- or underestimation of accumulation in later cycles where there could be a large difference in observed effect of toxicity between kidneys in one animal. The initial kidney pharmacokinetics of [^177^Lu]Lu-PSMA-617 were largely unchanged during dynamic scans in cycle 1 and 2 for both [^177^Lu]Lu-PSMA-617 groups. The exception was one kidney in a [^177^Lu]Lu-PSMA-617 + PBS animal with a drastically slower and higher accumulation in cycle 2. Initially thought to be an individual anomaly, however, we then observed the same pattern in an increased number of animals during cycle 3. Since it mathematically is an outlier value, absorbed dose calculations were done both including and excluding it. The delay in reaching maximum accumulation in the kidney, and the increase of that maximum, in later cycles seem to indicate an effect of nephrotoxicity. The post-cycle 3 imaging of [^111^In]In-PSMA-617 had a lower kidney accumulation for the PSMA groups at 25 min post injection than was observed with [^177^Lu]Lu-PSMA-617 during cycle 3, but this could be due to a further change in the accumulation curve. Our results show that at this level of therapy, the absorbed dose cannot be assumed from measurement during only a single cycle.

The final calculated kidney absorbed dose was lower for the group given rA1M, which might indicate a renoprotective effect for rA1M resulting from slower radioligand kinetics. However, since there were no significant differences between [^177^Lu]Lu-PSMA-617 and [^177^Lu]Lu-PSMA-617 + rA1M groups with regards to albumin in urine and serum BUN levels, and only four animals per group per cycle were imaged for dosimetry, this is not by itself conclusive evidence. When performing [^111^In]In-PSMA-617 imaging post cycle 3, the measurements at 7 h p.i. show that if excluding the three kidneys from rA1M animals labelled by the analysis software, using the Tukey method^31^, as major outliers, then there would be significantly (*p* = 0.015) more accumulation in the [^177^Lu]Lu-PSMA-617 + PBS group at that time, although then only 19 kidneys from 11 animals remain in the [^177^Lu]Lu-PSMA-617 + rA1M group for analysis. The measured lower kidney absorbed dose per injected MBq for the [^177^Lu]Lu-PSMA-617 + rA1M group in the third cycle while no significant differences between groups in the later [^111^In]In-PSMA-617 imaging or in biomarkers, could point to either uncertainties due to low statistics in dosimetry, or a radioprotective effect of rA1M up to two cycles which was then overwhelmed by the additional absorbed dose. Future research into the potential radioprotective effect of rA1M might be wise to focus on continuous measurements of kidney function after a larger amount of individually smaller treatment cycles. Our own experience of [^99m^Tc]Tc-MAG_3_ imaging after injection of 100 MBq [^177^Lu]Lu-PSMA-617 was that it yielded small effects^24^ but [^99m^Tc]Tc-DMSA imaging could be more practical and has been shown to be a good marker for kidney damage in rats after PRRT^32^.

The radiotoxic compound used in PRRT, [^177^Lu]Lu-DOTATATE, is filtrated from the blood in the glomeruli and reabsorbed in the proximal tubule. To avoid toxic side-effects on the kidneys during PRRT, an absorbed dose limit for humans of 23 Gy for both kidneys has been adapted following experience from external radiation therapy^33^. However, the radiobiological situation in radioligand therapy is radically different than external radiation therapy when it comes to absorbed dose rate, spatial absorbed dose distribution etc.^17^. Therefore, the absorbed dose limit is probably higher than 23 Gy. The tolerable absorbed doses in mice might also be different than in humans. A 2015 study by Haller et al*.*^34^ investigated kidney damage in mice after folate receptor-target RLT and found that using histological scoring, absorbed doses like those found for the [^177^Lu]Lu-PSMA-617 + rA1M group in this study, in the 45–50 Gy range, gave moderately severe renal damage. The higher of the values for the [^177^Lu]Lu-PSMA-617 + PBS group would fit into their results of > 69 Gy giving severe renal damage. We did not perform identical scoring but an overall histological analysis of our material, and counting of the number of glomeruli, showed less difference between [^177^Lu]Lu-PSMA-617 mice and untreated controls than the Haller et al*.* one. This could be due to differences in radiotoxicity due to the microscopic distribution of the radioligand, which for folate receptor targeting is similar to [^177^Lu]Lu-DOTATATE but might be different for [^177^Lu]Lu-PSMA-617, something that can also be observed in that renal radiotoxicity has so far not been a clinical concern for this ligand^35^.

[^177^Lu]Lu-PSMA-617 has specific uptake in kidney tissue via PSMA or a similar extracellular target^36,37^. The heterogeneity of the accumulation of [^177^Lu]Lu-PSMA-617 in the kidney will affect the absorbed dose distribution and probably the nephrotoxicity but due to the comparatively low spatial resolution of the SPECT data we did not investigate this further^38^.

rA1M has protective effects in several kidney-related toxicity models, including PRRT with [^177^Lu]Lu-DOTATATE^19,23^. It is not fully understood through which mechanism/-s rA1M act as a radioprotector although it has been suggested that rA1M utilizes its radical scavenging and reductase abilities to prevent oxidative stress-induced damage on healthy kidney tissue by reactive oxygen species resulting from radiolysis as well as dying cells^23,39^. Therefore, it is interesting that we did not detect any significant protective effects here with [^177^Lu]Lu-PSMA-617. One reason might be a difference in renal filtration between [^177^Lu]Lu-PSMA-617 and [^177^Lu]Lu-DOTATATE. However, PSMA-617 is an only slightly smaller molecule than DOTATATE, 1.2 vs 1.6 kDa^40^ so the filtration kinetics through the kidney should not differ substantially, and both are much smaller than A1M at 26 kDa with the recombinant forms being slightly smaller^41^. It might, however, be that the difference in pharmacokinetics and/or receptors in the kidneys would warrant another route of administration of rA1M in RLT with [^177^Lu]Lu-PSMA-617. In Alattar et al., s.c. and i.p. administration, in addition to i.v., was evaluated and it was concluded that i.v. was preferable in the PRRT model with [^177^Lu]Lu-DOTATATE. In humans, a continuous infusion over the course of hours might be beneficial, especially since the biological half-life in blood of A1M is quite short^18^. But to not inflict to much stress on the mice by constraining them for hours, we here instead gave them two injections (0 and 24 h p.i.), as previously described^23,24^, although it might have rendered different results with a continuous infusion instead.

Recently, rA1M was also reported to significantly reduce Major Adverse Kidney Events (MAKE) in patients undergoing cardiac surgery^42^, highlighting the clinical relevance of our study. However, it should be noted that rA1M had less effect in patients with inferior kidney function, which might be why we did not see any protective effect here; that is, the insult(s) might have damaged the animal kidneys beyond protection.

No nephrotoxicity was reported in the VISION clinical trial^10^. However, this trial was for patients with metastasized castration-resistant prostate cancer that have an overall survival measured in months post therapy. If radioligand therapy was to be administered at an earlier stage of the disease, it is possible that nephrotoxicity would become a greater concern, making radioprotection warranted^17^.

To conclude, we have shown that treating mice with three cycles of 200 MBq of [^177^Lu]Lu-PSMA-617 gives detectable and non-transient nephrotoxicity and hematotoxicity. The high activity, multi treatment cycle design may provide a template for future studies of different types of toxicity and interventions designed to mitigate them. This study did not show a significant protective effect of rA1M but there are some indications of a preservative effect on pharmacokinetics that should be further investigated.

## Methods

### Radiopharmaceuticals

Radiolabeling was performed similarly to previous work^27^. Freeze-dried PSMA-617 (MedChemExpress, Monmouth Junction, NJ, USA) was dissolved in chelexed 0.2 M ammonium acetate, pH 5.5, to a final concentration of 2 µg/µL. To the reaction solution containing 60 µg (58 nmol) PSMA-617, 107 μL (3860 MBq) of no-carrier-added ^177^LuCl_3_ (ITM, Garching bei München, Germany) was added and the final reaction volume was diluted to 350 µL using 0.2 M ammonium acetate, pH 5.5. The reaction vial was incubated at 90 °C for 60 min and then cooled at room temperature for 5 min. Thereafter 1 μL samples were taken for the analysis of the radiolabeling yield by ITLC SG (Agilent Technologies, Santa Clara, CA, USA) with 0.2 M citric acid buffer pH 2 as the mobile phase. To reduce radiolysis associated with high radioactivity concentrations, the final solution was diluted with a solution of 2% Bovine Serum Albumin (BSA) in Phosphate Buffered Saline (PBS) containing 100-fold molar excess EDTA (Ethylenediaminetetraacetic acid disodium salt dihydrate). The final volume was 100 µL (201 MBq) per injected dose. The purity of the final solution was further confirmed using instant thin-layer chromatography.

Preparation of [^111^In]In-PSMA-617 was performed under the same labeling conditions of pH and temperature. In brief, 185 MBq of ^111^InCl_3_ was incubated with 60 µg (58 nmol) PSMA-617 for 60 min before the mixture was allowed to cool down and 1 μL samples were analyzed using ITLC SG. The final solution was diluted with a solution of 2% BSA in PBS containing 100-fold molar excess EDTA.

### In vivo studies

**Figure 7:**
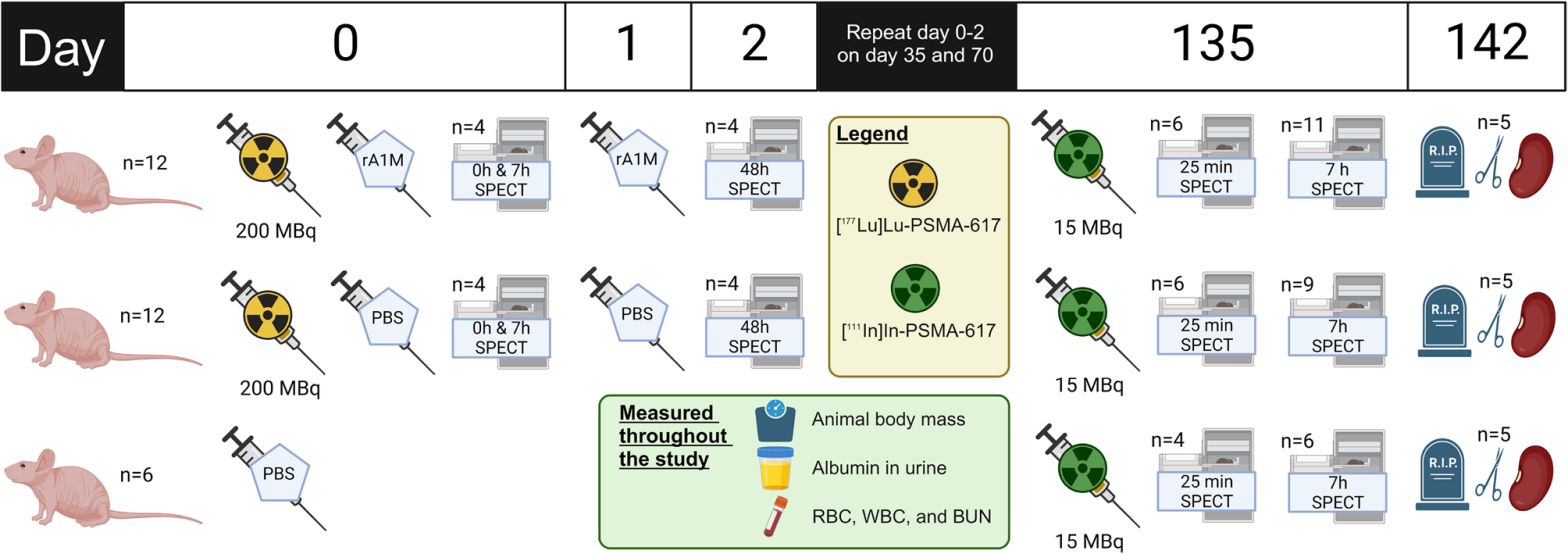
Scheme of in vivo experiments. Time schedule for the main treatments of the three animal groups.

Male BALB/cAnNRj nude mice (Janvier Labs, Le Genest-Saint-Isle, France) were used for in vivo studies. All Animals were given ad libitum access to food and water. An overview of the in vivo studies can be found in Fig. 7.

Every 5 weeks, animals in the [^177^Lu]Lu-PSMA-617 groups were injected i.v. first with either 5 mg/kg (approx. 30 µL) rA1M (Guard Therapeutics International AB, Stockholm, Sweden, n = 12) or 30 µL PBS (n = 12) and then directly with approximately 200 MBq, 3.0 nmol, 100 µL [^177^Lu]Lu-PSMA-617 with repeated administration of rA1M or corresponding volume of PBS again 24 h later. One group was only injected with PBS (control, n = 6). The activity of the ^177^Lu syringe was measured before and after injection to record the correct injected activity. Animals were given a total of three cycles (approximately 600 MBq). Animals were weighed throughout the study and all animals were sacrificed after 142 days post first injection.

An additional group of 6 animals were given 50 MBq, 0.8 nmol, 25 µL [^177^Lu]Lu-PSMA-617 each i.v. Three of these were sacrificed at 2 weeks p.i. and three at 3 weeks p.i. Kidneys were excised, weighed and the activity measured in a well counter (2480 Wizard^2^ automatic, PerkinElmer, Waltham, MA, USA) to calculate percent of injected activity per gram.

### Dosimetry and radioligand accumulation

For every cycle, 4 animals from the [^177^Lu]Lu-PSMA-617 + rA1M group and 4 animals from the [^177^Lu]Lu-PSMA-617 + PBS group were selected for imaging for dosimetry. No animal was selected for more than one cycle. These animals were anesthetized with 2–3% Isoflurane (Baxter, Deerfield, IL, USA) in a O_2_ and N_2_O mix, placed in the animal bed of the SPECT/CT (NanoSPECT/CT Plus, Mediso; Budapest, Hungary), and immediately a CT scan was performed. The field-of-view for SPECT imaging was set to the location of the kidneys on the CT image. Injections were performed, and SPECT imaging started approximately half a minute post ^177^Lu injection on a protocol of dynamic imaging with 90–100 frames of 1 min length each. A 1-h static SPECT image of the whole animal was also acquired at 7 h p.i. and at 48 h p.i. For the first cycle, an additional static SPECT image was taken at 96 h p.i. but it was calculated that this could be substituted by correcting the final mean absorbed dose per injected activity (Gy/MBq) result by a factor of 0.9019 when only using 7 and 48 h p.i. static scans, introducing only a mean error of 1.2%. Also, during the first cycle, a standard of known ^177^Lu activity was included in the field-of-view to ensure that the high injected activity did not affect the detector efficiency. All SPECT images were reconstructed using HiSPECT software (SciVis, Göttingen, Germany) and the “Standard” pre-set.

SPECT images were quantitatively analysed using VivoQuant 3.0 software (inviCRO, Boston, MA, USA). Any activity measured on the static scans as remaining in the tail of the animal was subtracted from the injected activity when calculating accumulation in each image, and decay corrected from the 7 h p.i. scan to also be included for calculations of the dynamic scan.

Due to the limited axial field of view, the dynamic scan did not always include both kidneys so either one or two volumes of interest were manually drawn in those images and in the ones from the static scans. For each quantified kidney, a graph of percentage of injected activity per gram (assuming that 1 cm^3^ = 1 g) was created by linear interpolation between SPECT datapoints, as well as the biodistribution values for 2 and 3 weeks p.i., and the assumption of no accumulation at zero seconds post injection. The final linear interpolation was then forward-projected to 5 weeks post injection and the whole graph decay-corrected to calculate the number of ^177^Lu decays in the kidney over 5 weeks. The number of decays up to 48 h p.i. was also calculated. The total decays were then converted to the absorbed dose over 5 weeks by multiplication with a kidney-to-kidney ^177^Lu S value from the MOBY mouse phantom based Monte Carlo calculation of Larsson et al.^43^ and the kidney weight used in that simulation, 0.21082 g. In the case both kidneys were measured for an animal, these two values were averaged into one. The absorbed doses were divided by injected activity for each animal and averaged per group to reach a mean absorbed dose per activity (Gy/MBq) value which was then employed on all animals in that group. In the second cycle, one outlier kidney with a very high accumulation was identified in the [^177^Lu]Lu-PSMA-617 + PBS group and calculations were made both with and without this kidney included.

At 135 days post the first injection, due to the observed differences in radioligand uptake in the administration of the third cycle, all surviving animals, [^177^Lu]Lu-PSMA-617 + rA1M, [^177^Lu]Lu-PSMA-617 + PBS and untreated animals, where injected with 15.4 ± 0.1 MBq, 4.8 nmol, 100 µL of [^111^In]In-PSMA-617. Two static SPECT imaging sessions of 15- and 60-min length, centred around 25- and 420-min p.i. were performed. For the 25 min time point, 6 animals of each PSMA group were imaged, and 4 untreated animals. And for the 420 min time point, 11 [^177^Lu]Lu-PSMA-617 + rA1M, 9 [^177^Lu]Lu-PSMA-617 + PBS and 6 untreated animals were imaged. Volumes of interest were drawn for both kidneys on all images and percent injected accumulation per gram calculated.

### Histology

Assessments of normal glomerular formations were performed in one sagittal section at the center position of the kidneys. Post animal sacrifice and excision, kidneys were dehydrated in a graded alcohol series ended with Xylen (100%) and were embedded in paraffin. Sectioning was performed with a rotating microtome and adjacent sections (4 μm) were collected on microscope slides (SuperFrost plus slides; Merck) from the middle portion of the kidney, yielding three to four sections per slide. Slides were heated to 60 °C for one hour and paraffin was removed by immersion in Xylen, and sections were rehydrated via immersion in EtOH (100%, 96% and 70%) followed by water. Sections were stained with hematoxylin (Mayers HTX Plus; Histolab, Gothenburg, Sweden), followed by incubation in sodium bicarbonate (0,012 M) and 70% EtOH, and were then stained with eosin solution (Eosin B; Applichem, GmbH, Darmstadt, Germany). Slides were then dehydrated in EtOH (96% and 100%), followed by xylene (100%). Sections were mounted in mounting medium (Pertex; Histolab, Gothenburg, Sweden). Standard bright-field microscope analyses were performed by two histologists, performing manual counting of glomerular formations of all sections.

### Blood cell and platelet count

Blood was sampled (every 1–3 weeks) after injections. Samples (20 µL) were collected from the tail vein or vena saphena from awake, immobilized mice by piercing the vein with a needle and collecting blood in a K2EDTA-coated plastic micropipette (Boule Medical, Stockholm, Sweden). Total white blood cell counts (WBC, 10^9^/L), lymphocyte counts (LYM, 10^9^/L), monocyte counts (MONO, 10^9^/L), granulocyte counts (GRAN, 10^9^/L), hemoglobin (HBG, g/dL), hematocrit (HCT, %), red blood cell counts (RBC, 10^12^/L), mean cell volume (MCV, femtoliter = fL) and platelets (PLT, 10^9^/L) were measured in an Exigo Veterinary (Exigo Vet) Hematology Analyzer (Boule Medical).

### Biomarkers of kidney damage

Urine was sampled (every 2–4 weeks) throughout the study and stored at − 80 °C. Albumin concentrations in urine were analyzed using a mouse albumin simple-step ELISA kit (Abcam, Cambridge, UK) according to manufacturer instructions. The QuantiChrom™ Creatinine Assay Kit assay was performed according to the manufacturer’s instructions (DICT-500, BioAssay Systems, Hayward, CA, USA) to establish the albumin/creatinine ratio. Serum was sampled 2–3 weeks from vena saphena (Microvette® CB 300 Serum, Sarstedt, Germany) after each cycle and before sacrifice (6 weeks after third cycle) and blood urea nitrogen (BUN) was analyzed with the Urea Nitrogen (BUN) Colorimetric Detection Kit (Invitrogen, Waltham, Massachusetts, USA) according to the manufacturer’s instructions.

### Statistical analysis

Statistical calculations were performed with GraphPad Prism (GraphPad Prism 9.4; GraphPad Software; GraphPad, Bethesda, MD, USA) or with SPSS Statistics version 27 (IBM, Armonk, NY, USA). Statistical tests are specified in the respective figure legends, and only significant differences are presented in the figures. Significance requires *p* < 0.05.

## Ethics approval

All animal experiments in this study were approved by the Malmö/Lund Animal Experimentation Ethics Committee at the Lund district court (Dnr: 04,350–2020 with the addition Dnr 5.8.18–07,300/2021) and conducted in compliance with the ARRIVE guidelines and other relevant guidelines and regulations.

### Supplementary Information

Supplementary Figures.

## Data Availability

The datasets used and/or analyzed during the current study are available from the lead contact on reasonable request.

## References

[1] Sung H (2021). Global cancer statistics 2020: GLOBOCAN estimates of incidence and mortality worldwide for 36 cancers in 185 countries. CA A Cancer J. Clin..

[2] Tolkach Y (2018). Prostate-specific membrane antigen in breast cancer: a comprehensive evaluation of expression and a case report of radionuclide therapy. Breast Cancer Res. Treat..

[3] Rahbar K (2017). German multicenter study investigating 177Lu-PSMA-617 radioligand therapy in advanced prostate cancer patients. J.Nucl. Med..

[4] Harsini S (2021). A prospective study on [68Ga]-PSMA PET/CT imaging in newly diagnosed intermediate-and high-risk prostate cancer. Asia Ocean. J. Nucl. Med. Biol..

[5] Uprimny C (2017). 68 Ga-PSMA-11 PET/CT in primary staging of prostate cancer: PSA and Gleason score predict the intensity of tracer accumulation in the primary tumour. Eur. J. Nucl. Med. Mol. Imag..

[6] Bravaccini S (2018). PSMA expression: a potential ally for the pathologist in prostate cancer diagnosis. Sci. Rep..

[7] Hofman MS (2018). [177Lu]-PSMA-617 radionuclide treatment in patients with metastatic castration-resistant prostate cancer (LuPSMA trial): a single-centre, single-arm, phase 2 study. Lancet Oncol..

[8] Emmett L (2019). Results of a prospective phase 2 pilot trial of 177Lu–PSMA-617 therapy for metastatic castration-resistant prostate cancer including imaging predictors of treatment response and patterns of progression. Clin. Genitourin. Cancer.

[9] Heitkötter B (2018). Neovascular PSMA expression is a common feature in malignant neoplasms of the thyroid. Oncotarget.

[10] Sartor O (2021). Lutetium-177–PSMA-617 for metastatic castration-resistant prostate cancer. N. Engl. J. Med..

[11] Chang SS, Reuter VE, Heston W, Gaudin PB (2001). Metastatic renal cell carcinoma neovasculature expresses prostate-specific membrane antigen. Urology.

[12] Ahlstedt J (2015). Biodistribution and pharmacokinetics of recombinant α1-microglobulin and its potential use in radioprotection of kidneys. Am. J. Nucl. Med. Mol. Imag..

[13] Schäfer H (2023). Extensive 177Lu-PSMA radioligand therapy can lead to radiation nephropathy with a renal thrombotic microangiopathy–like picture. Eur. Urol..

[14] Rao DV, Howell RW (1993). Time-dose-fractionation in radioimmunotherapy: Implications for selecting radionuclides. J. Nucl. Med. Off. Publ. Soc. Nucl. Med..

[15] Groener D (2021). Hematologic safety of 177Lu-PSMA-617 radioligand therapy in patients with metastatic castration-resistant prostate cancer. EJNMMI Res..

[16] Emami B (1991). 1991 Three-dimensional treatment planning for lung cancer. Int. J. Radiat. Oncol. * Biol. * Phys..

[17] Wahl RL (2021). Normal-tissue tolerance to radiopharmaceutical therapies, the knowns and the unknowns. J. Nucl. Med..

[18] Bergwik J, Kristiansson A, Allhorn M, Gram M, Åkerström B (2021). Structure, functions, and physiological roles of the lipocalin α1-microglobulin (A1M). Front. Physiol..

[19] Kristiansson A (2019). Protection of kidney function with human antioxidation protein α1-microglobulin in a mouse 177Lu-DOTATATE radiation therapy model. Antioxidants Redox Signal..

[20] Andersson CK (2019). Recombinant α1-microglobulin is a potential kidney protector in 177Lu-octreotate treatment of neuroendocrine tumors. J. Nucl. Med..

[21] Wester L, Michaelsson E, Holmdahl R, Olofsson T, Akerström B (1998). Receptor for alpha1-microglobulin on T lymphocytes: Inhibition of antigen-induced interleukin-2 production. Scand. J. Immunol..

[22] Kristiansson A (2021). Human radical scavenger α1-microglobulin protects against hemolysis in vitro and α1-microglobulin knockout mice exhibit a macrocytic anemia phenotype. Free Radical Biol. Med..

[23] Alattar AG (2023). Recombinant α1-Microglobulin (rA1M) protects against hematopoietic and renal toxicity, alone and in combination with amino acids, in a 177Lu-DOTATATE mouse radiation model. Biomolecules.

[24] Kristiansson A (2021). 177Lu-PSMA-617 therapy in mice, with or without the antioxidant α1-microglobulin (A1M), including kidney damage assessment using 99mTc-MAG3 imaging. Biomolecules.

[25] Geenen L (2021). Overcoming nephrotoxicity in peptide receptor radionuclide therapy using [177Lu]Lu-DOTA-TATE for the treatment of neuroendocrine tumours. Nucl. Med. Biol..

[26] Kristiansson A (2021). Kidney protection with the radical scavenger α1-microglobulin (A1M) during peptide receptor radionuclide and radioligand therapy. Antioxidants.

[27] Kristiansson A (2022). Hematological toxicity in mice after high activity injections of 177Lu-PSMA-617. Pharmaceutics.

[28] Wysoczynski M (2017). Poor mobilization in T-cell-deficient nude mice is explained by defective activation of granulocytes and monocytes. Cell Transpl..

[29] Tschan VJ (2022). Preclinical investigations using [(177)Lu]Lu-Ibu-DAB-PSMA toward its clinical translation for radioligand therapy of prostate cancer. Eur. J. Nucl. Med. Mol. Imag..

[30] Boutaleb S (2009). Impact of mouse model on preclinical dosimetry in targeted radionuclide therapy. Proc. IEEE.

[31] Tukey, J. W. Exploratory Data Analysis (Addison-Wesley, 1977).

[32] Forrer F (2007). From outside to inside? Dose-dependent renal tubular damage after high-dose peptide receptor radionuclide therapy in rats measured with in vivo 99mTc-DMSA-SPECT and molecular imaging. Cancer Biother. Radiopharm..

[33] Park, E. A., Graves, S. A. & Menda, Y. The Impact of Radiopharmaceutical Therapy on Renal Function. *Semi. Nucl. Med.* **52**, 467–474. 10.1053/j.semnuclmed.2022.02.004 (2022). 10.1053/j.semnuclmed.2022.02.004 35314056

[34] Haller S (2015). Folate receptor-targeted radionuclide therapy: Preclinical investigation of anti-tumor effects and potential radionephropathy. Nucl. Med. Biol..

[35] Herrmann K (2024). Renal and multiorgan safety of <sup>177</sup>Lu-PSMA-617 in patients with metastatic castration-resistant prostate cancer in the VISION dosimetry substudy. J. Nucl. Med..

[36] Lucaroni L (2023). Cross-reactivity to glutamate carboxypeptidase III causes undesired salivary gland and kidney uptake of PSMA-targeted small-molecule radionuclide therapeutics. Eur. J. Nucl. Med. Mol. Imag..

[37] Lee Z, Heston WD, Wang X, Basilion JP (2023). GCP III is not the “off-target” for urea-based PSMA ligands. Eur. J. Nucl. Med. Mol. Imag..

[38] Saldarriaga Vargas, C. *et al.* Heterogeneity of absorbed dose distribution in kidney tissues and dose–response modelling of nephrotoxicity in radiopharmaceutical therapy with beta-particle emitters: A review. Zeitschrift für Medizinische Physik. 10.1016/j.zemedi.2023.02.006 (2023).10.1016/j.zemedi.2023.02.00637031068

[39] Olsson MG (2010). Bystander cell death and stress response is inhibited by the radical scavenger α1-microglobulin in irradiated cell cultures. Radiat. Res..

[40] Kim S (2016). PubChem substance and compound databases. Nucl. Acids Res..

[41] Kwasek A (2007). Production of recombinant human α1-microglobulin and mutant forms involved in chromophore formation. Protein Expr. Purif..

[42] *Guard Therapeutics reports robust efficacy of RMC-035 in Phase 2 (AKITA) and advances clinical development program*, <https://guardtherapeutics.com/en/press-releases/guard-therapeutics-reports-robust-efficacy-of-rmc-035-in-phase-2-akita-and-advances-clinical-development-program> (2023).

[43] Larsson E, Ljungberg M, Strand S-E, Jönsson B-A (2011). Monte Carlo calculations of absorbed doses in tumours using a modified MOBY mouse phantom for pre-clinical dosimetry studies. Acta Oncol..

