# Supplementary material for: Hematological and renal toxicity in mice after three cycles of high activity [177Lu]Lu-PSMA-617 with or without human α1-microglobulin
Source: Sci Rep. 2024 May 11;14:10787. doi: 10.1038/s41598-024-61370-2 (PMC11088679; doi:10.1038/s41598-024-61370-2)
Supplement: Supplementary file 1 — Supplementary Figures. [file 41598_2024_61370_MOESM1_ESM.pdf]

Supplementary material

Hematological and renal toxicity in mice after three cycles of high activity [^{177}Lu]Lu-PSMA-617 with or without human α_1-microglobulin

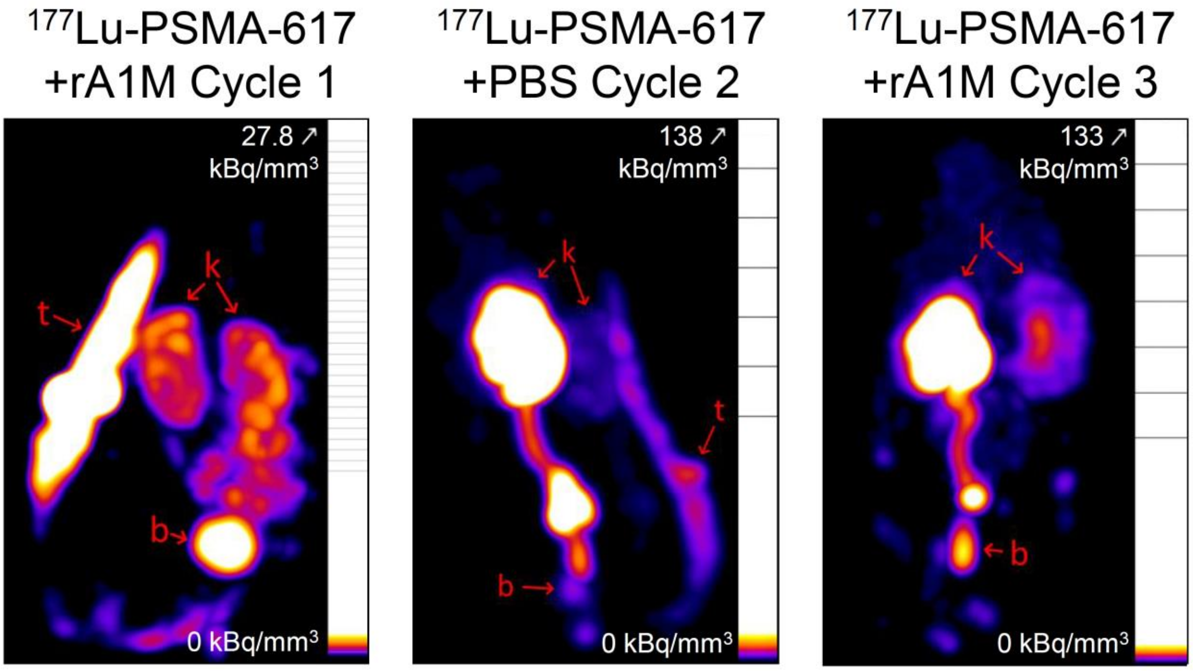

FIGURE S1. Uptake characteristics of [^{177}Lu]Lu-PSMA-617 changes after each cycle of treatment. Representative SPECT images and graphs of [^{177}Lu]Lu-PSMA-617 activity in male BALB/cAnNRj nude mice . Maximum intensity projections using the NIH color scale windowed to best display kidneys with the top activity noted. Note that the Cycle 2 animal is the one with an outlier kidney. Annotations: b = bladder, k = kidney, and t=tail. Static 1 hour scan at 7 h p.i. of the same animals as in Figure 1, convolved by a 2 mm FWHM Gauss filter. Chosen animals have typical distribution for their cycles except for the second cycle animal where one kidney is an outlier.

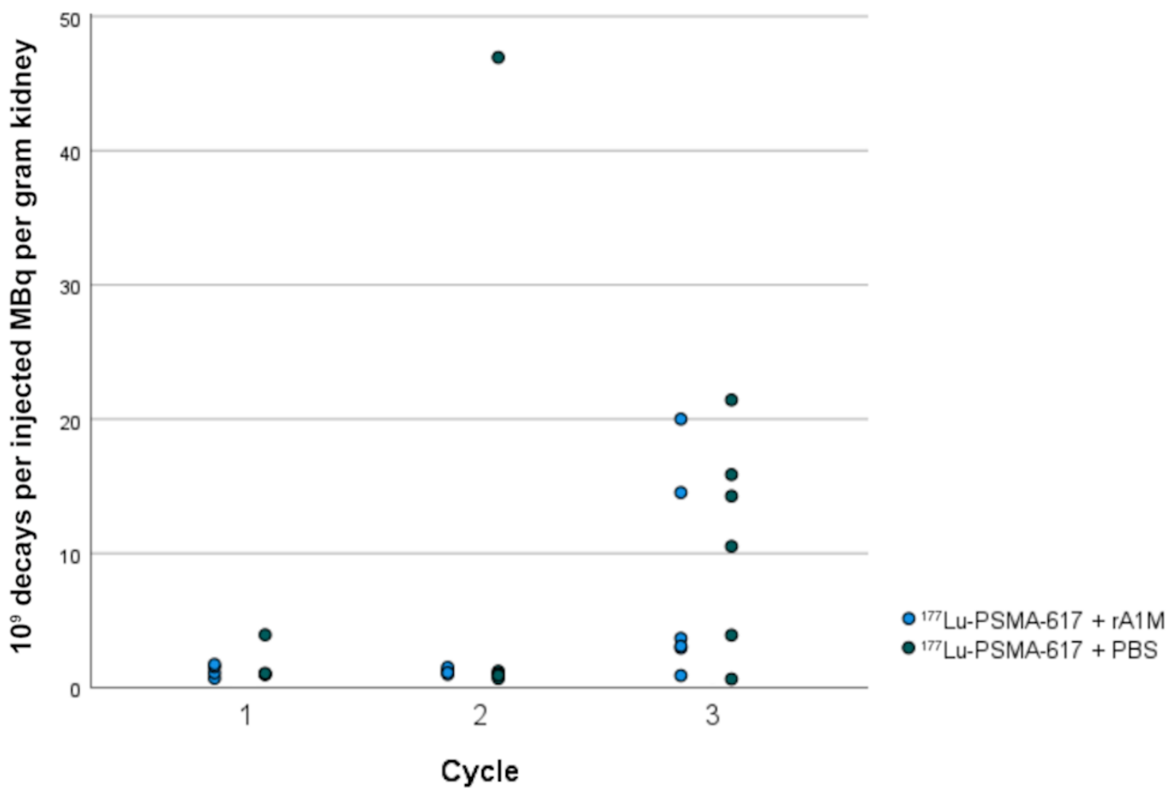

FIGURE S2. Total radioactive decays in the kidney changes after each cycle of treatment. Number of decays calculated in measured kidneys (different animals each cycle) for the first 48 hours per injected activity. Note the outlier [^{177}Lu]Lu-PSMA-617 + PBS kidney in cycle 2

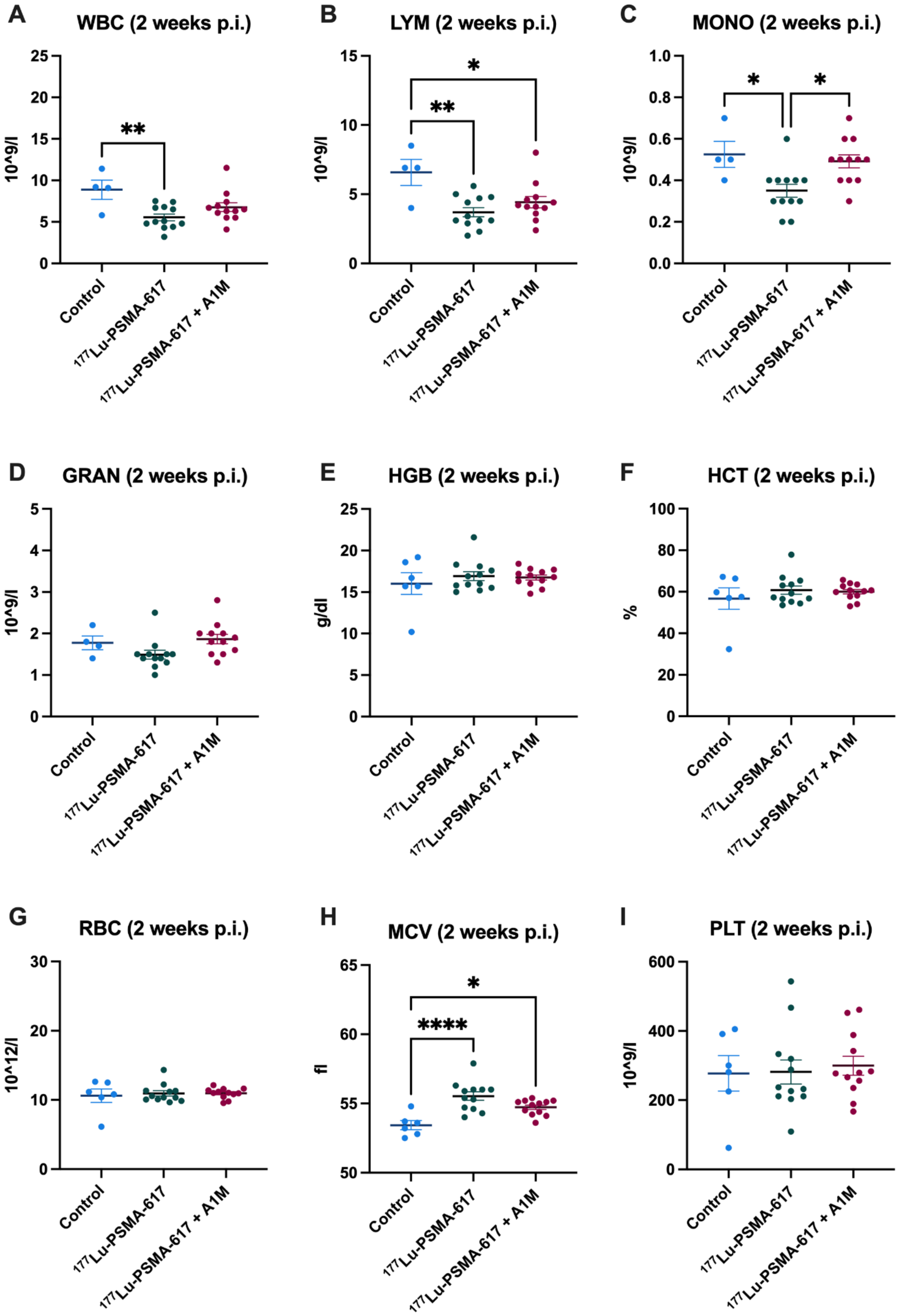

FIGURE S3. Blood cell counts two weeks post-injection 1. WBC (A), LYM (B), MONO (C), GRAN (D), HGB (E), HCT (F), RBC (G), MCV (H) and PLT (I). Data are presented scatter plots with mean (± SEM). Statistical Statistical comparison between groups was made with one-way ANOVA with a Tukey's multiple comparisons post hoc test (A–I). Only significant differences are presented in the figure. *$p < 0.05$, ** $p < 0.01$., *** $p < 0.001$, **** $p < 0.0001$.

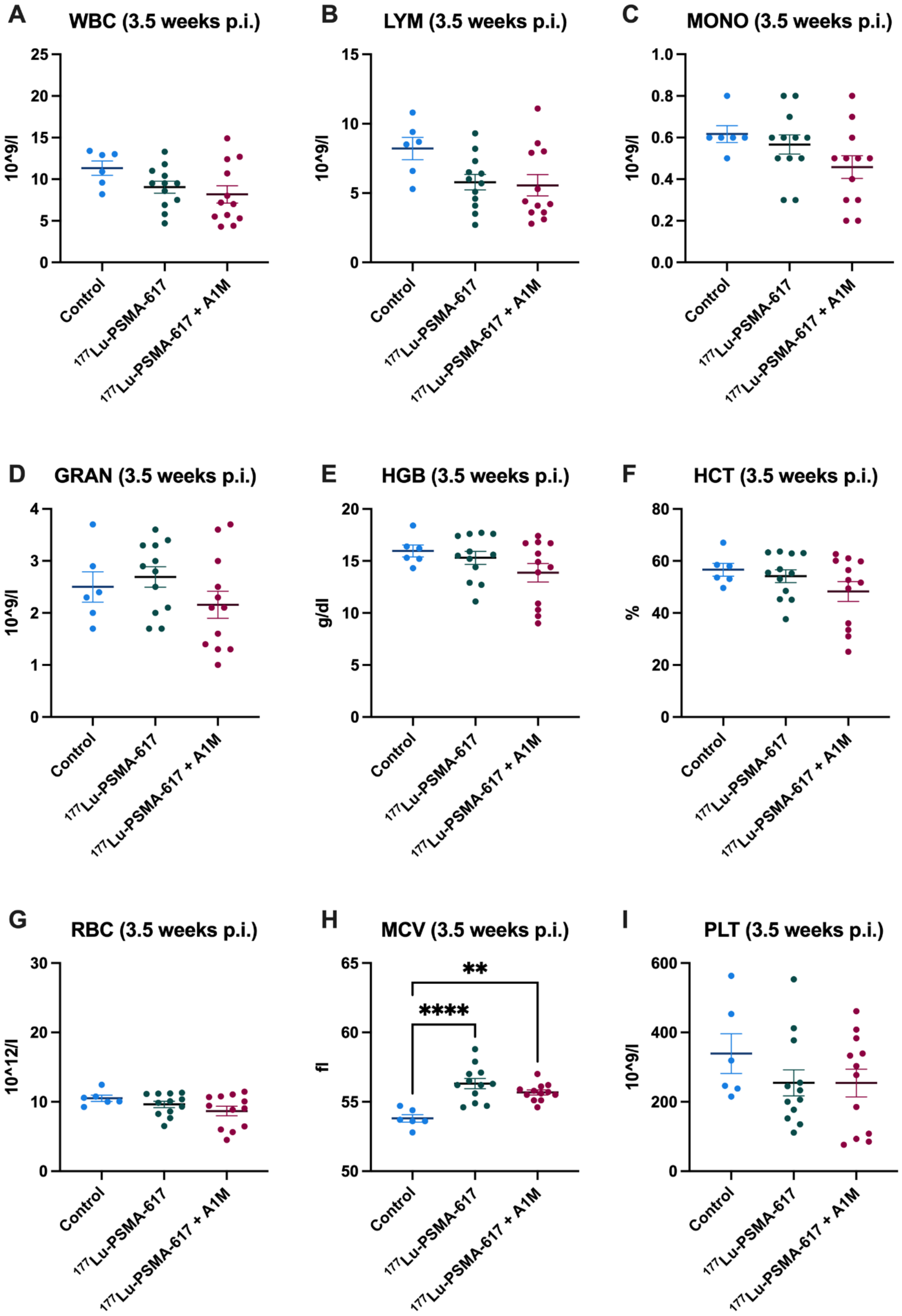

FIGURE S4. Blood cell counts 3.5 weeks post-injection 1. WBC (A), LYM (B), MONO (C), GRAN (D), HGB (E), HCT (F), RBC (G), MCV (H) and PLT (I). Data are presented scatter plots with mean (± SEM). Statistical Statistical comparison between groups was made with one-way ANOVA with a Tukey's multiple comparisons post hoc test (A–I). Only significant differences are presented in the figure. *$p < 0.05$, ** $p < 0.01$., *** $p < 0.001$, **** $p < 0.0001$.

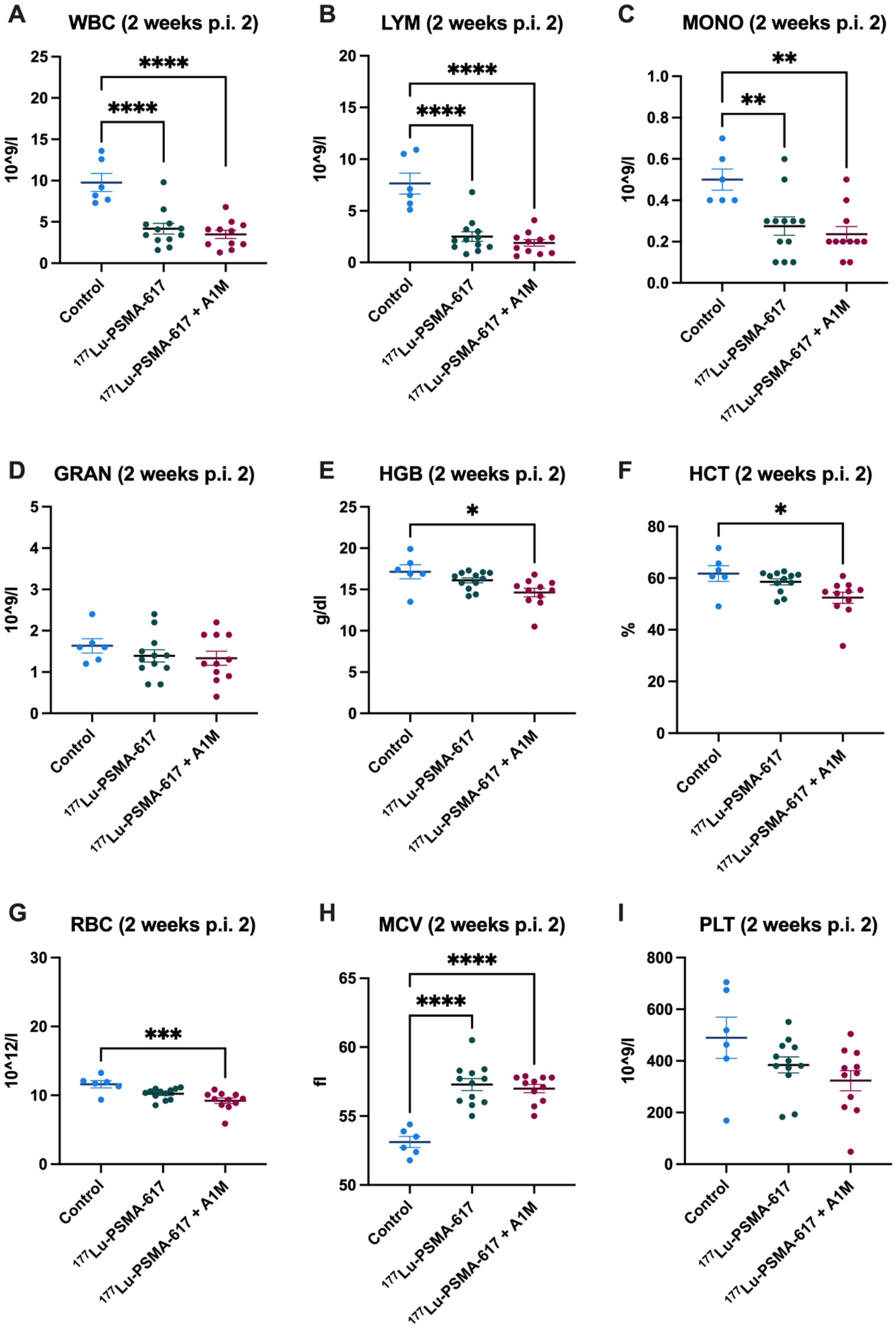

FIGURE S5. Blood cell counts two weeks post-injection 2. WBC (A), LYM (B), MONO (C), GRAN (D), HGB (E), HCT (F), RBC (G), MCV (H) and PLT (I). Data are presented scatter plots with mean (± SEM). Statistical comparison between groups was made with one-way ANOVA with a Tukey's multiple comparisons post hoc test (A–I). Only significant differences are presented in the figure. *$p < 0.05$, ** $p < 0.01$., *** $p < 0.001$, **** $p < 0.0001$.

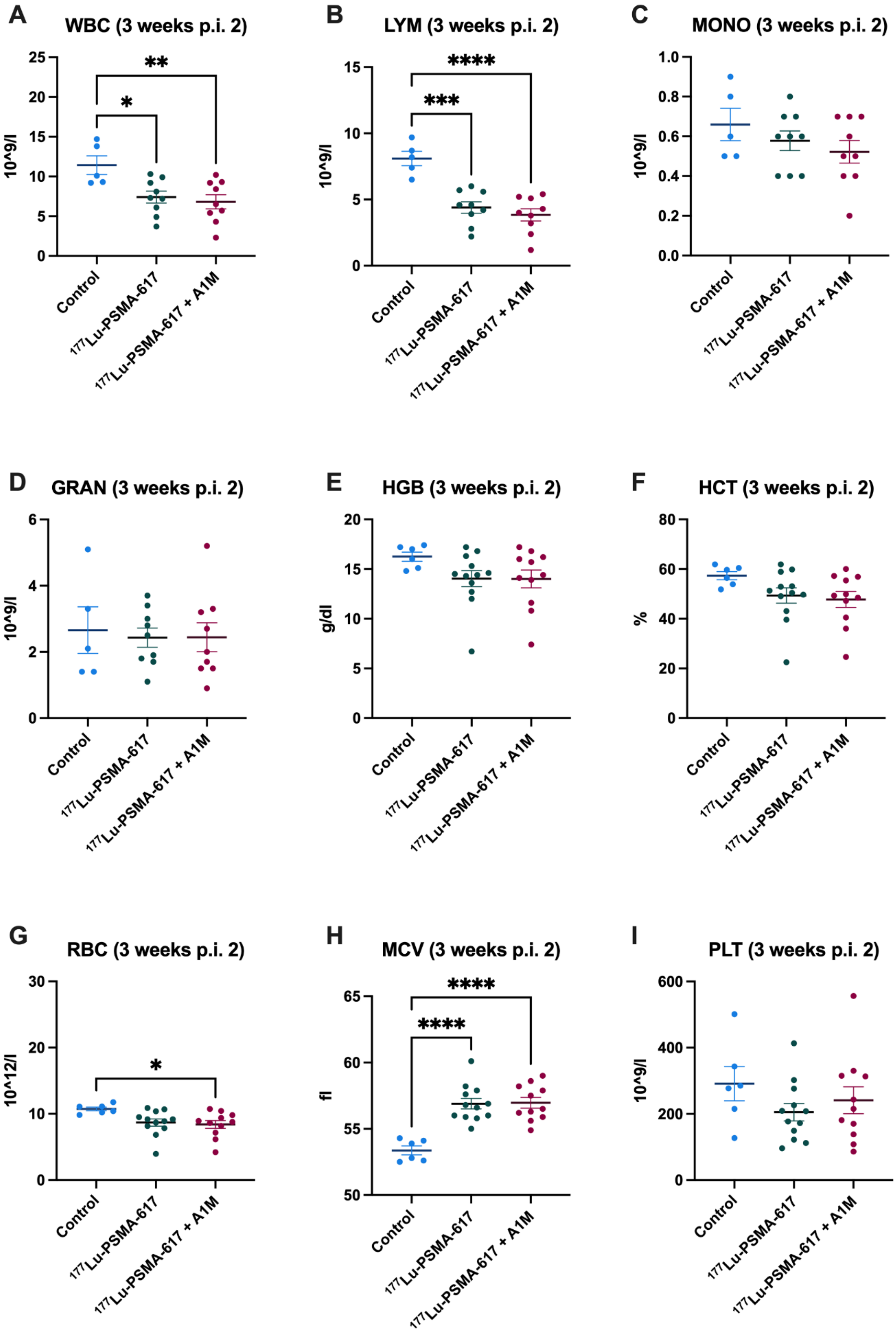

FIGURE S6. Blood cell counts three weeks post-injection 2. WBC (A), LYM (B), MONO (C), GRAN (D), HGB (E), HCT (F), RBC (G), MCV (H) and PLT (I). Data are presented scatter plots with mean (± SEM). Statistical Statistical comparison between groups was made with one-way ANOVA with a Tukey's multiple comparisons post hoc test (A–I). Only significant differences are presented in the figure. *$p < 0.05$, ** $p < 0.01$., *** $p < 0.001$, **** $p < 0.0001$.

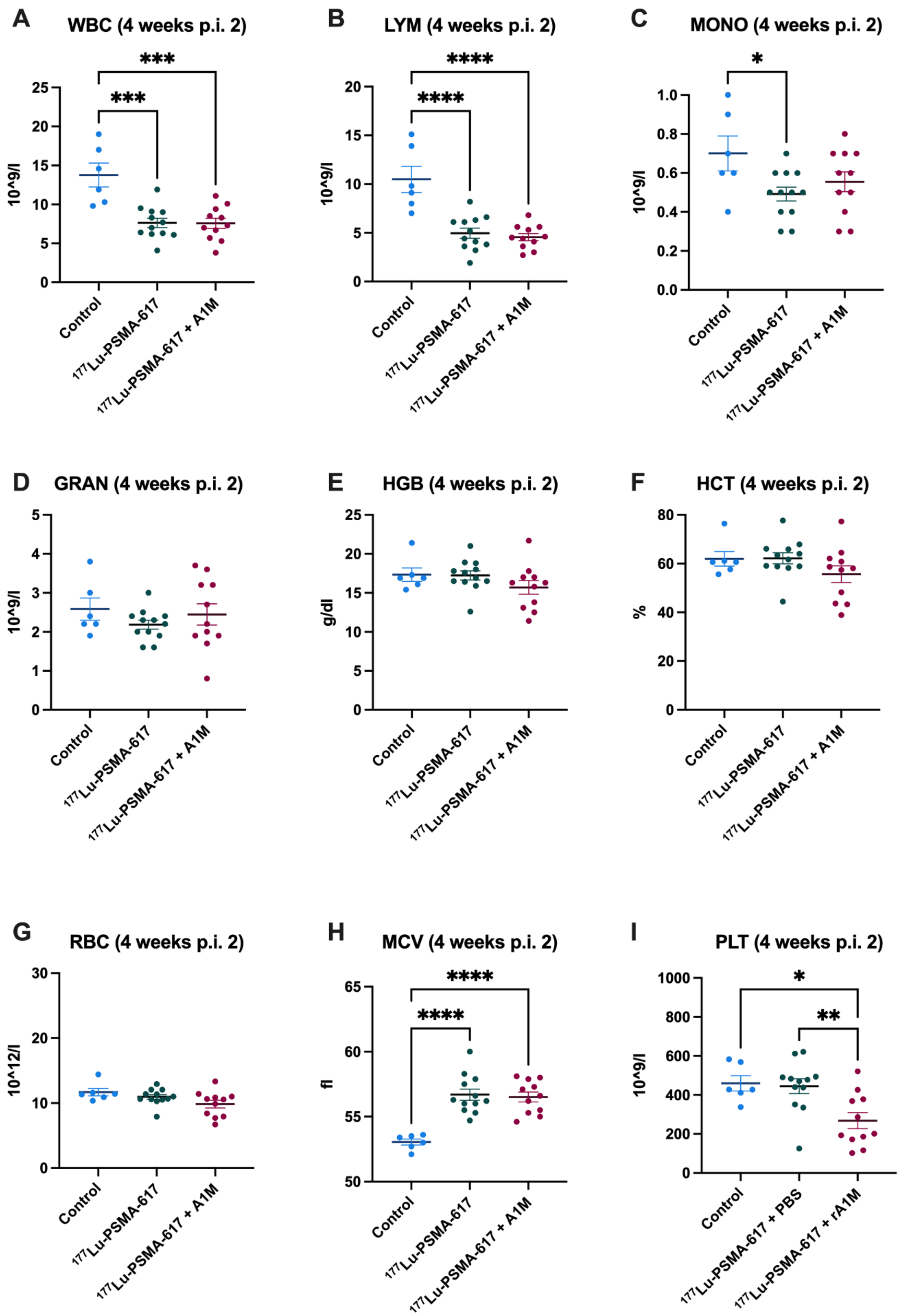

FIGURE S7. Blood cell counts four weeks post-injection 2. WBC (A), LYM (B), MONO (C), GRAN (D), HGB (E), HCT (F), RBC (G), MCV (H) and PLT (I). Data are presented scatter plots with mean (± SEM). Statistical Statistical comparison between groups was made with one-way ANOVA with a Tukey's multiple comparisons post hoc test (A–I). Only significant differences are presented in the figure. *$p < 0.05$, ** $p < 0.01$., *** $p < 0.001$, **** $p < 0.0001$.

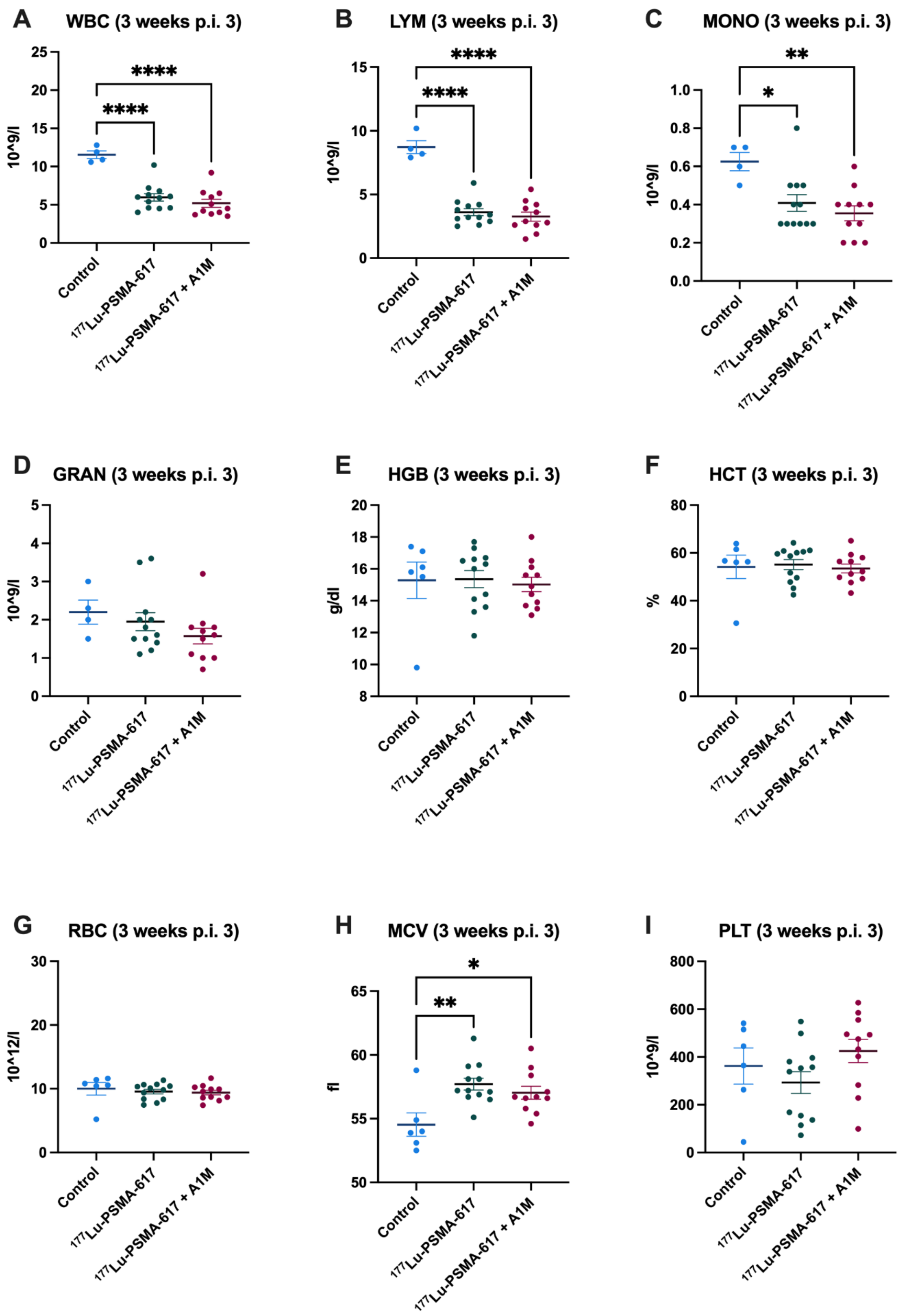

FIGURE S8. Blood cell counts three weeks post-injection 3. WBC (A), LYM (B), MONO (C), GRAN (D), HGB (E), HCT (F), RBC (G), MCV (H) and PLT (I). Data are presented scatter plots with mean (± SEM). Statistical Statistical comparison between groups was made with one-way ANOVA with a Tukey's multiple comparisons post hoc test (A–I). Only significant differences are presented in the figure. *$p < 0.05$, ** $p < 0.01$., **** $p < 0.001$, **** $p < 0.0001$.*

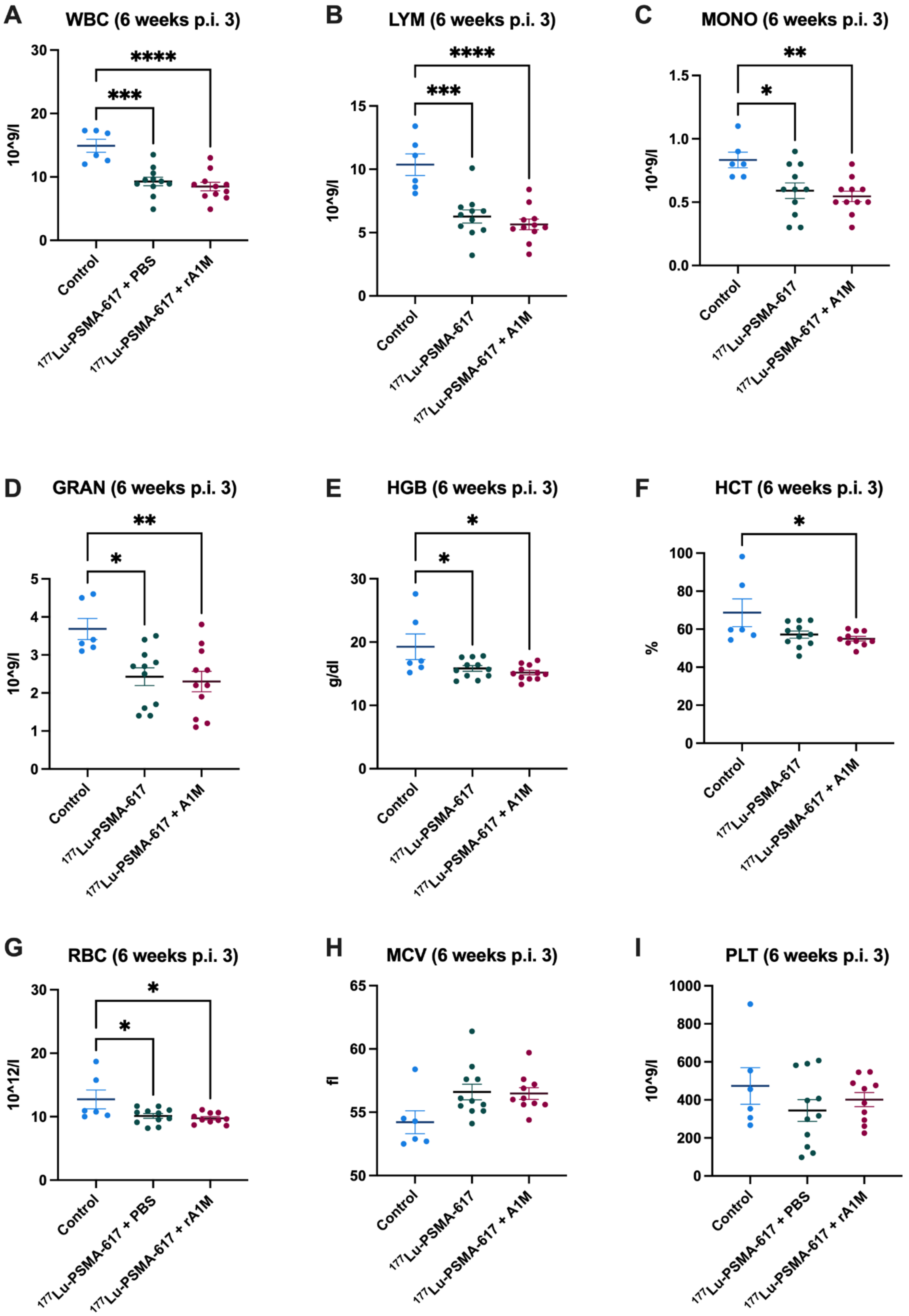

FIGURE S9. Blood cell counts six weeks post-injection 3. WBC (A), LYM (B), MONO (C), GRAN (D), HGB (E), HCT (F), RBC (G), MCV (H) and PLT (I). Data are presented scatter plots with mean (± SEM). Statistical Statistical comparison between groups was made with one-way ANOVA with a Tukey's multiple comparisons post hoc test (A–I). Only significant differences are presented in the figure. *$p < 0.05$, ** $p < 0.01$., *** $p < 0.001$, **** $p < 0.0001$.

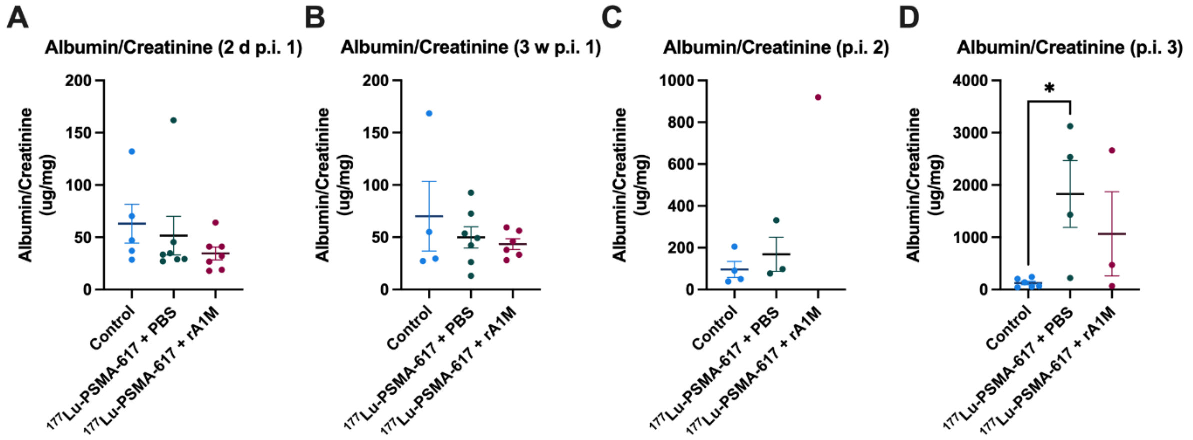

FIGURE S10. Animals treated with [^{177}Lu]Lu-PSMA-617 had elevated albumin/creatinine ratio in urine. Albumin/creatinine levels in urine two days after first cycle (A), three weeks after first cycle (B), after second cycle (C), and after third cycle (D). Data are presented as scatter plots with mean (± SEM). Statistical Statistical comparison between groups was made with one-way ANOVA with a Tukey's multiple comparisons post hoc test (A, B and D) and with a student t-test (C) between control and [^{177}Lu]Lu-PSMA-617 + PBS group. Only significant differences are presented in the figure. *$p < 0.05$

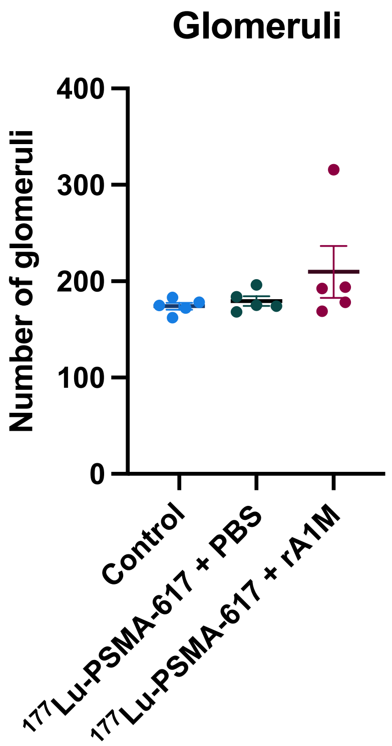

FIGURE S11. Number of functional glomeruli in the kidneys after three fractions of [^{177}Lu]Lu-PSMA-617. Ordinary one-way ANOVA with Tukey's multiple comparisons test. Statistical Statistical comparison between groups was made with one-way ANOVA with a Tukey's multiple comparisons post hoc test.
